# Fluoranthene-based dopant-free hole transporting materials for efficient perovskite solar cells†
†Electronic supplementary information (ESI) available. CCDC 1589253, 1589258 and 1589261. For ESI and crystallographic data in CIF or other electronic format see DOI: 10.1039/c7sc05484j


**DOI:** 10.1039/c7sc05484j

**Published:** 2018-02-02

**Authors:** Xianglang Sun, Qifan Xue, Zonglong Zhu, Qi Xiao, Kui Jiang, Hin-Lap Yip, He Yan, Zhong’an Li

**Affiliations:** a Key Laboratory for Material Chemistry of Energy Conversion and Storage , Ministry of Education , School of Chemistry and Chemical Engineering , Huazhong University of Science and Technology , 430074 , Wuhan , P. R. China . Email: lizha@hust.edu.cn; b Department of Chemistry , Energy Institute and Hong Kong Branch of Chinese National Engineering Research Center for Tissue Restoration and Reconstruction , The Hong Kong University of Science and Technology , Clear Water Bay , Kowloon , Hong Kong , China . Email: zzhuab@connect.ust.hk ; Email: hyan@ust.hk; c Institute of Polymer Optoelectronic Materials and Devices , State Key Laboratory of Luminescent Materials and Devices , South China University of Technology , 510006 , Guangzhou , P. R. China

## Abstract

Significant efforts have been devoted to developing new dopant-free hole transporting materials (HTMs) for perovskite solar cells (PVSCs). Fluoranthene is one typical cyclopentene-fused polycyclic aromatic hydrocarbon with a rigid planarized structure, and thus could be an ideal building block to construct dopant-free HTMs, which have not been reported yet. Here, we report a new and simple synthetic method to prepare unreported 2,3-dicyano-fluoranthene through a Diels–Alder reaction between dibenzofulvene and tetracyanoethylene, and demonstrate that it can serve as an efficient electron-withdrawing unit for constructing donor–acceptor (D–A) type HTMs. This novel building block not only endows the resulting molecules with suitable energy levels, but also enables highly ordered and strong molecular packing in solid states, both of which could facilitate hole extraction and transport. Thus with dopant-free HTMs, impressive efficiencies of 18.03% and 17.01% which are associated with enhanced stability can be achieved based on conventional n–i–p and inverted p–i–n PVSCs respectively, outperforming most organic dopant-free HTMs reported so far.

## Abstract 2

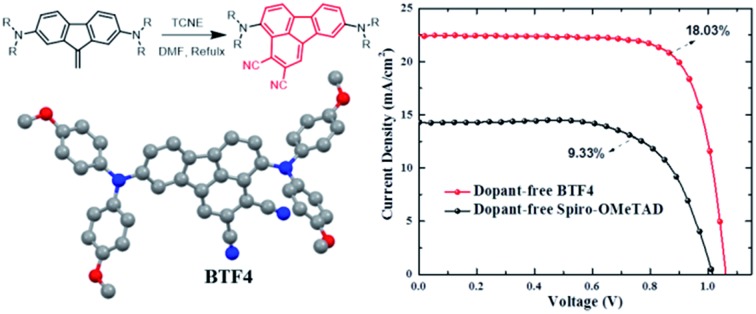
Unreported 2,3-dicyano-fluoranthene was first prepared as an efficient electron-withdrawing building block for constructing D–A type dopant-free hole transporting materials.

## Introduction

Organic–inorganic hybrid perovskite solar cells (PVSCs) have triggered worldwide attention due to their impressive research progress within a short time and their very promising market prospects.^1^–^5^ Recently, the record-high power conversion efficiency (PCE) of PVSCs has reached the certified value of 22%, almost rivaling that of the crystalline silicon based photovoltaics.^6^ When fabricating PVSCs, the introduction of suitable interfacial materials, *i.e.* electron transporting materials (ETMs) and hole transporting materials (HTMs), is critical for achieving high device performance, because they not only improve the charge carrier transport/collection efficiency, but also act as a protective layer for perovskites to enhance device stability.^7^–^11^

For HTMs applied in PVSCs, organic semiconductors are more popular over their inorganic counterparts which is attributed to their milder processing conditions that are compatible with perovskites. Nevertheless, most organic HTMs exhibit relatively low hole mobility, and thus need to be improved through a chemical doping process by ionic dopants such as Li–bis(trifluoromethanesulfonyl)imide (Li–TFSI).^12^–^16^ However, the doping process always induces a negative effect on device stability due to the sophisticated oxidation process associated with undesired ion migration/interactions.^17^,^18^ For example, the device PCE derived from the well-known doped HTM, 2,2′,7,7′-tetrakis(*N*,*N*-bis(*p*-methoxyphenyl)amino)-9,9′-spirobifluorene (spiro-OMeTAD), often vanished after 30 days of ambient storage, even though the maximum of its initial PCE was close to 19%.^19^ Thus, development of efficient dopant-free HTMs is urgently needed. However few dopant-free HTMs can show comparable PCEs to the doped spiro-OMeTAD.^18^,^20^–^26^

Normally, HTMs would not need an additional doping process if they exhibit hole mobility up to 10^–4^–10^–3^ cm^2^ V^–1^ s^–1^.^27^ To this end, two molecular design strategies, donor–acceptor (D–A) type and star-shaped structure, have been mainly used for designing dopant-free HTMs with better intermolecular interactions to ensure sufficient hole mobility.^17^,^28^–^34^ By integrating these two strategies, Nazeeruddin *et al.* prepared a new class of star-shaped D–A molecules that served as dopant-free HTMs towards ∼19% PCE with enhanced device stability.^19^,^35^ Nonetheless, most of the reported D–A type HTMs come from relatively complicated scaffolds with multistep syntheses and purification processes. We recently reported a new dipolar chromophore based dopant-free HTM *via* a facile synthesis, which afforded a high PCE of 16.9%.^36^ Following this success, it is crucial to exploit new D–A combinations towards high performance dopant-free HTMs with low synthetic complexity.

One of the key factors for designing high performance HTMs is designing a suitable core structure. Fluoranthene is one typical cyclopentene-fused polycyclic aromatic hydrocarbon, which exhibits a rigid planarized structure that can favor enhanced π–π stacking. More importantly, fluoranthene, as a substructure of fullerene, shows a typical electron-deficient character due to the cyclopenta-fused non-alternant structure.^37^–^40^ Thus, fluoranthene may be an ideal electron-withdrawing building block to construct D–A type HTMs for PVSCs, which have not yet been reported, possibly due to a lack of efficient molecular design strategy. In this study, we describe a new and facile synthetic method to prepare an unreported 2,3-dicyano-fluoranthene moiety as an efficient core towards high performance dopant-free HTMs.

## Results and discussion

**Scheme 1:**
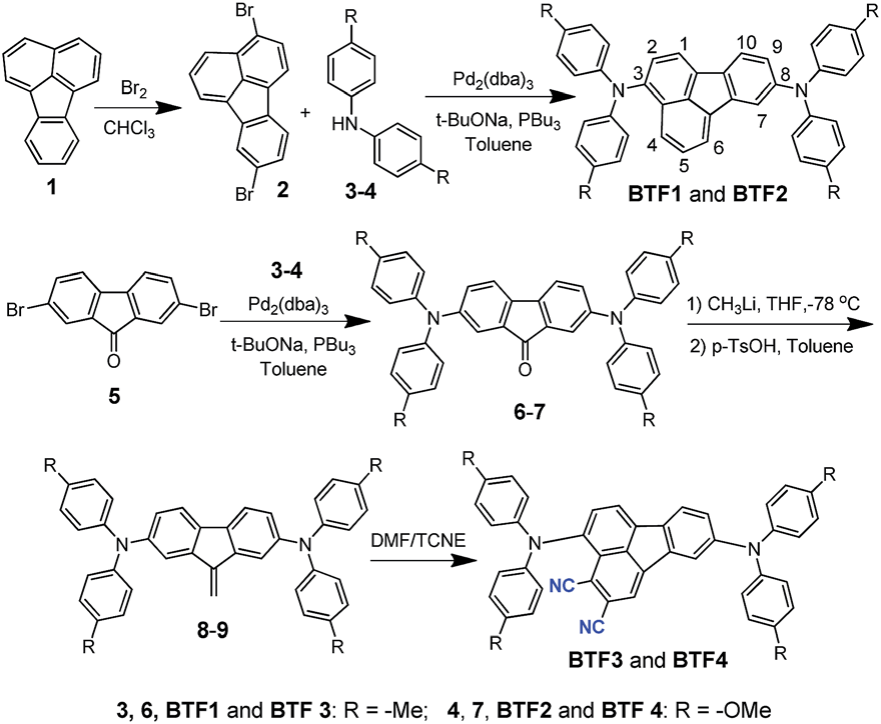
Synthetic route for fluoranthene-cored HTMs (**BTF1–4**).

**Scheme 2:**
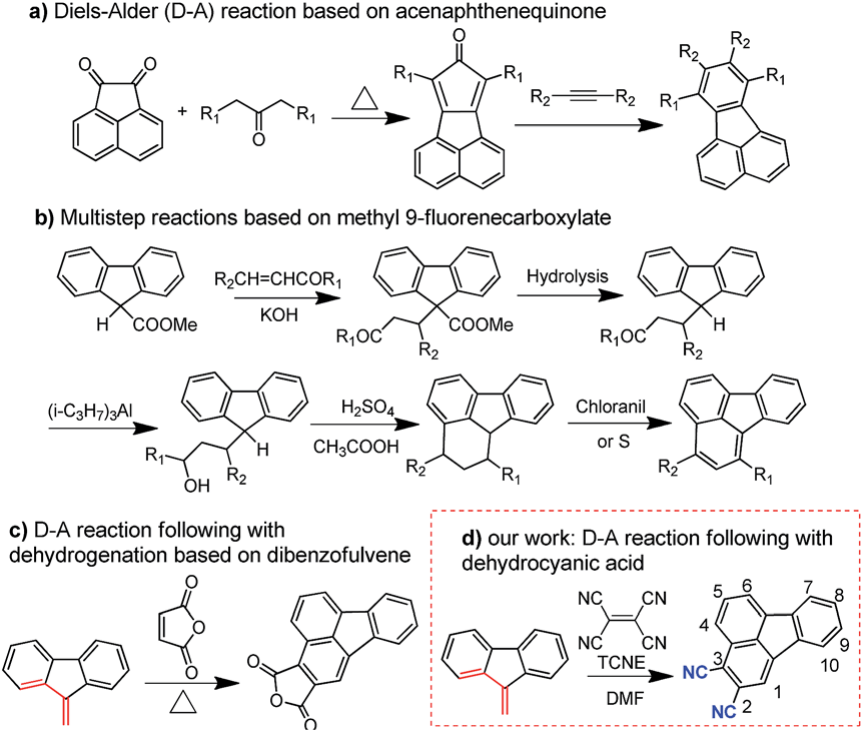
Synthetic methods for functionalized fluoranthenes.

The synthetic route of the HTMs is shown in Scheme 1, while the details are provided in the Experimental section in the ESI.† First, we prepared fluoranthene-cored **BTF1–2***via* a simple Buchwald–Hartwig coupling reaction between the readily accessible 3,8-dibromo-fluoranthene (**2**)^41^ and diphenylamine units (**3–4**). So far, facile synthesis of functionalized fluoranthene derivatives remains a challenge. One of the most widely used methods of affording fluoranthenes is through a typical Diels–Alder (D–A) reaction based on acenaphthenequinone derivatives (Scheme 2a), but the functionalized positions of this method are always limited, mainly to the C7–C10 positions.^42^,^43^ Another typical method proposed by Stubbs and Tucker *et al.* employs fluorene derivatives as the starting materials (Scheme 2b), but the total synthetic efficiency is too poor due to the tedious multistep reactions.^44^

Through careful searching, we encouragingly found that in 1949, dibenzofulvene and maleic anhydride were reported as the starting materials by Wang *et al.* to yield 2,3-substituted fluoranthene based on the D–A reaction followed by dehydrogenation (Scheme 2c).^45^ Although the yield of this reaction is low, only ∼10%, the simple and facile procedure is very attractive for us. Thus, with a purpose of synthesizing a more electron-deficient fluoranthene core, tetracyanoethylene (TCNE) was selected as the dienophile to react with dibenzofulvene (Scheme 2d) through the D–A reaction but followed by dehydrocyanic acid. Excitingly, 2,3-dicyano-fluoranthene was successfully obtained as we expected, in spite of a low isolated yield of 3.5%. With an indirect method, we prepared diphenylamine moieties substituted on dibenzofulvene (**8–9**) first, and then used them as the critical intermediates to react with TCNE as shown in Scheme 1, which successfully obtained our desired products, **BTF3–4**, with enhanced isolated yields (29.1% for **BTF3** and 43.0% for **BTF4**). The electron-rich substitutions were found to activate the reactivity of fulvene. This can be further evidenced by the higher yield of **BTF4**, as methoxyl groups in **9** make diene more electron-rich. Moreover, due to the facile synthesis, all synthetic costs, 7.1 $ g^–1^, 11.4 $ g^–1^, 53.1 $ g^–1^ and 62.8 $ g^–1^ for **BTF1–4** (Tables S1–S4†) respectively, are much lower than that of spiro-OMeTAD, making them good material candidates suitable for large scale production.

**Fig. 1:**
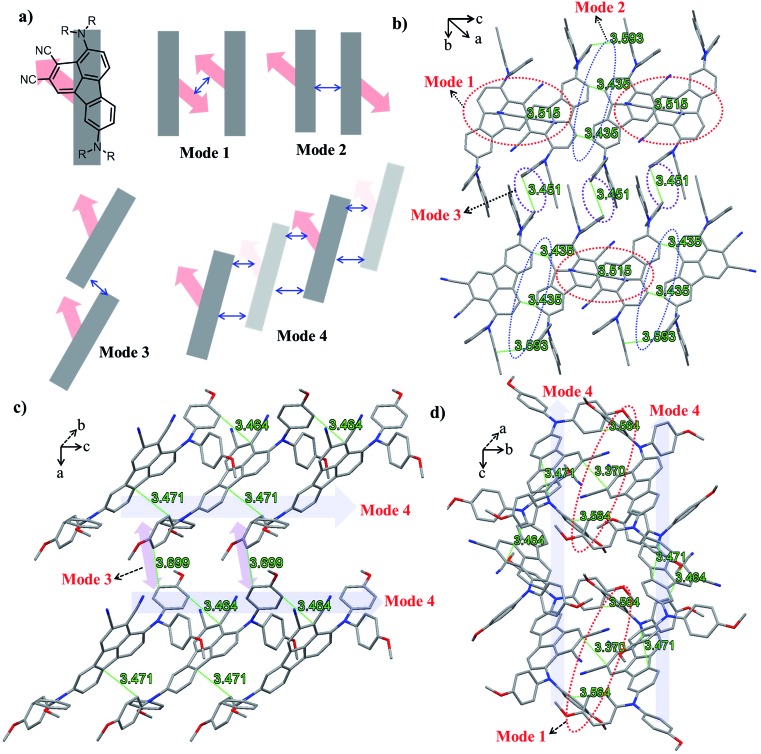
(a) Schematic representations of the π-stacking modes of **BTF3–4** based on single-crystal X-ray analyses, where block arrows show the direction of dipole moments, and double arrow lines represent the positions of π-stacking; (b) π-stacks of **BTF3** viewed along the *a* axis; (c) π-stacks of **BTF4** viewed along the *b* axis; and (d) π-stacks of **BTF4** viewed along the *a* axis. For clarity, the hydrogen atoms and solvent molecules in **BTF3** are omitted. The π-stacking distances are noted in green.

The ground-state geometric structures of **BTF3** (triclinic, *P*1 space group) and **BTF4** (monoclinic, *P*2_1_/*c* space group) were refined using X-ray crystallography, and the detailed data are shown in Tables S5–S6† in the ESI.† Fig. 1 shows the packing arrangements of **BTF3–4**. According to the relative arrangement between molecules and contact positions, four types of molecular packing mode are found (Fig. 1a and S3–S4†), including H-type dimeric stacking through dipole–dipole interactions due to dicyano-substitutions (mode 1), H-type dimeric stacking through π–π interactions between two adjacent molecules (mode 2), J-type dimeric stacking through π–π interactions between two adjacent diphenylamine units (mode 3), and herringbone stacking through π–π interactions between two adjacent molecules (mode 4).

As shown in Fig. 1b, **BTF3** molecules pack into highly ordered and extended H-aggregates along the (0–11) plane, wherein two types of molecular stacking (mode 1 and 2) appear in an alternating fashion. In stacking mode 1, the stacking distance between two dicyano-fluoranthene units is 3.515 Å, while that in stacking mode 2 is 3.435 Å. Except for short stacking distance, the contact area in mode 2 is much larger than that in mode 1, as the diphenylamine units also show a close contact with each other with a distance of 3.593 Å. Moreover, it is encouragingly found that **BTF3** molecules in two adjacent H-aggregates can pack into highly ordered J-like aggregates along the (100) plane based on stacking mode 3 with a π–π-distance of 3.451 Å. Thus, the solid state structure of **BTF3** can be classified as a two-dimensional “brick-wall”-like assembly.

**Fig. 2:**
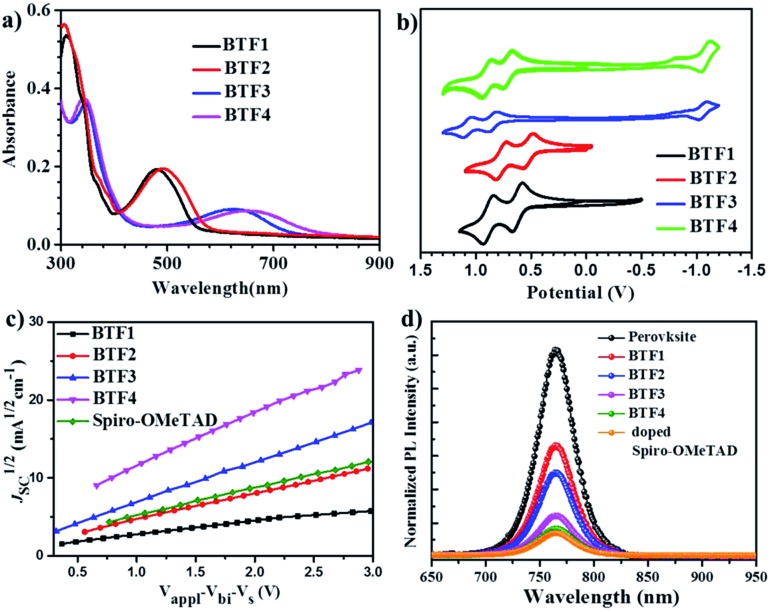
(a) The film absorption spectra. (b) The CV curves *versus* Fe/Fe^+^ (0.66 V) measured in DCM solution. (c) The hole injection characteristics measured by the SCLC method based on the device structure of ITO/V_2_O_5_/HTMs/V_2_O_5_/Al. (d) Steady state PL spectra of the bilayered perovskite films with or without capping with different HTLs.

**BTF4** molecules interestingly show a highly ordered herringbone assembly with partial edge-to-face packing along the (101) plane (stacking mode 4, Fig. 2c). Due to the asymmetric nature of cyano-fluoranthene, they make contact in three different positions (Fig. S4†) with a π–π-distance of 3.464 Å, 3.471 Å, and 3.617 Å respectively, making the molecular packing very dense. Differently from those observed in **BTF3**, the neighboring herringbone assemblies interact with each other along both the (110) and (101) planes, based on stacking mode 3 and 1 respectively. Thus, the packing structure of **BTF4** can be classified as a quasi-three-dimensional herringbone assembly. The edge-to-face π–π-distance within the dimer in stacking mode 3 for **BTF4** is slightly longer (3.669 Å) than that in **BTF3**. However, a much shorter dipole–dipole interaction distance of 3.37 Å is obtained for **BTF4** in stacking mode 4, attributed to the close edge-to-face π-contact with a distance of 3.564 Å between diphenylamine units that also occurs. In contrast, we only obtained a low-quality single crystal of **BTF1** for structure analysis, which indicates an absence of strong π–π interactions (Fig. S5†). Based on these results, we can conclude that cyano-substitution plays a critical role in enabling strong intermolecular interactions in solid states, which is expected to be beneficial for achieving efficient charge transport.

The absorption spectra of **BTF1–4** in diluted solutions and as thin films are shown in Fig. S6† and 2a respectively, with related data listed in Table 1. All spectra show two distinct absorption bands, ascribed to localized π–π* transitions and intramolecular charge transfer (ICT). By comparing the film spectra with their corresponding solution spectra, it is found that the low-energy absorption bands of **BTF3–4** are more red-shifted than those of **BTF1–2**, suggesting that aggregation of the former is more pronounced, which is consistent with the results from the crystal structure analysis. Both methoxy- and cyano-substitutions result in a red-shift in the absorption bands, while the red-shift degree for the latter is much larger, up to 150 nm of the absorption maximum for **BTF4**, ascribed to more effective ICT. The absorption edges of **BTF1–4** in thin films are determined to be 554, 575, 731 and 781 nm respectively, corresponding to the optical bandgaps (*E*_opt_, Table 1) of 2.24, 2.16, 1.70, and 1.59 eV respectively.

**Table 1:** Optical, electrochemical, and hole mobility properties of **BTF1–4**

HTMs	*λ*_sol_ (nm)^*a*^	*λ*_fil_ (nm)^*b*^	*E*_opt_ (eV)^*c*^	*E*_HOMO_ (eV)^*d*^	*E*_LUMO_ (eV)	*μ* (cm^2^ V^–1^ s^–1^)^*f*^
**BTF1**	304 475	310 481	2.24	–4.94	–2.68^*e*^	1.46 × 10^–5^
**BTF2**	305 482	308 493	2.16	–4.80	–2.59^*e*^	2.13 × 10^–5^
**BTF3**	344 609	347 629	1.70	–5.19	–3.46^*d*^	6.36 × 10^–5^
**BTF4**	344 632	344 654	1.59	–5.02	–3.38^*d*^	1.17 × 10^–4^

^*a*^Absorption maxima in DCM solutions.

^*b*^Absorption maxima of thin films.

^*c*^Optical band gap calculated from film absorption edges.

^*d*^Measured from electrochemistry experiments.

^*e*^Calculated by an equation of *E*_LUMO_ = *E*_HOMO_ + *E*_opt_.

^*f*^Measured by SCLC method.

The highest occupied molecular orbital (HOMO) and lowest unoccupied molecular orbital (LUMO) levels were determined by cyclic voltammetry (CV), and the CV curves in DCM solutions are given in Fig. 2b, with data summarized in Table 1. **BTF3–4** display reversible oxidative and reductive processes, while only oxidative processes are observed for **BTF1–2**. The HOMO levels of **BTF1–4** are obtained as –4.94, –4.80, –5.19, and –5.02 eV respectively, *versus* Fc/Fc^+^. As shown, the cyano-substitution results in a decrease in the HOMO levels for **BTF3–4**, making them more compatible with perovskites to facilitate hole extraction (Fig. S10 and S17†). Moreover, we noted that the introduction of electron-rich methoxy groups can induce an increase in the HOMO levels. The LUMO levels of **BTF3–4** are calculated from the onset of reduction potential, –3.46 and –3.38 eV respectively, while those of **BTF1–2** are determined as –2.68 and –2.59 eV respectively, by subtracting *E*_opt_ from the HOMO levels.

The hole mobilities (*μ*) of **BTF1–4** without adding any dopants were measured by the space-charge-limited-current (SCLC) method (Fig. 2c). As shown in Table 1, the *μ* values of **BTF3–4** are 6.36 × 10^–5^ and 1.17 × 10^–4^ cm^2^ V^–1^ s^–1^respectively, much higher than those of **BTF1–2** and spiro-OMeTAD (2.36 × 10^–5^ cm^2^ V^–1^ s^–1^). The high mobility of **BTF4** would fulfill the needs of dopant-free HTMs. This can be attributed to its quasi-three-dimensional supramolecular assembly associated with much closer molecular stacking. We also characterized the steady-state photoluminescence (PL) spectra (Fig. 2d) of bilayered perovskite/non-doped HTL films to check their ability to extract holes. As seen, the PL of all bilayered films can be quenched by the introduction of hole transporting layers (HTLs), most effectively for **BTF4**, followed by **BTF3**, **BTF2** and then **BTF1**. It is worth noting that the quenching effectiveness of dopant-free **BTF3–4** is even comparable to that of doped spiro-OMeTAD (Fig. 2d), suggesting that holes are extracted efficiently from perovskites for **BTF3–4** even without adding any dopants.

We fabricated conventional n–i–p planar devices with a configuration of FTO/SnO_2_/PCBM/mixed perovskite/HTL/MoO_3_/Au to evaluate the efficacy of **BTF1–4** as dopant-free HTMs. Mixed perovskite (FAPbI_3_)_0.85_(MAPbBr_3_)_0.15_ (FA: NH = CHNH_3_^+^; MA: CH_3_NH_3_^+^) was used as the active light-harvesting layer. The device fabrication details are described in the ESI.† The morphology of **BT1–4** thin films on perovskites has been studied through scanning electron microscopy (SEM), and the images are shown in Fig. S11.† As can be clearly seen, all HTLs possess a smooth surface without pin-holes.

**Fig. 3:**
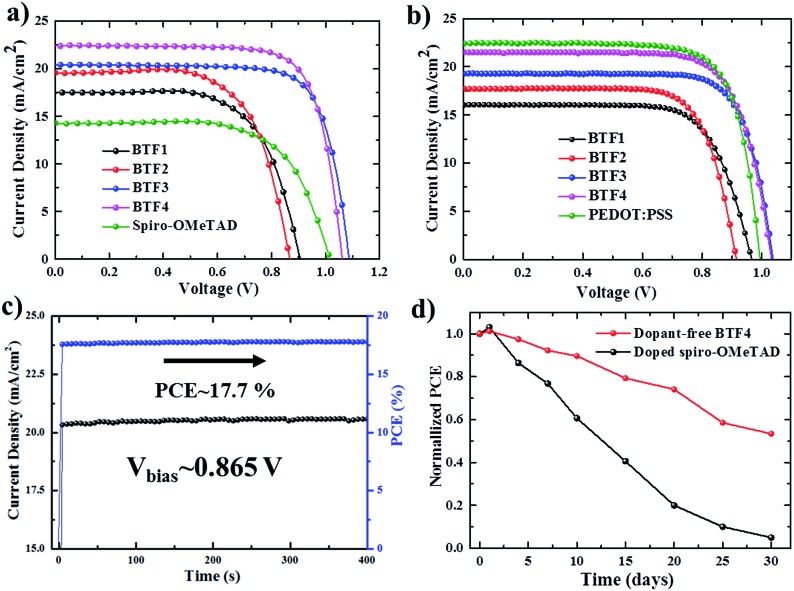
*J*–*V* curves of the best-performing conventional (a) and inverted (b) PVSCs with different dopant-free HTMs. (c) Stable output current of a dopant-free **BTF4** based conventional PVSC under a constant bias of 0.865 V and (d) the stability test of the conventional PVSCs in ambient air with a humidity of 40–50%.

The current density–voltage (*J*–*V*) curves of the best-performing conventional PVSCs using dopant-free HTMs are shown in Fig. 3a, with related photovoltaic parameters listed in Table 2. The champion devices based on dopant-free **BTF1** and **BTF2** showed limited PCEs of 9.97% and 10.45% respectively, which are only slightly enhanced compared to that of spiro-OMeTAD-based dopant-free control devices (9.33%) under the same fabrication conditions. In contrast, the device performance of dopant-free **BTF3–4** can be significantly improved, due to their more suitable HOMO levels and enhanced hole mobilities. The champion PVSC based on dopant-free **BTF4** delivered a very impressive PCE of 18.03% with an open-circuit voltage (*V*_oc_) of 1.06 V, a short-circuit photocurrent (*J*_sc_) of 22.5 mA cm^–2^, and a FF of 75.6%, which is among the best for dopant-free devices reported so far.^25^ For **BTF3**, its dopant-free devices exhibited a slightly decreased device PCE of 16.34%, mainly due to a reduction in the *J*_sc_ value caused by its inferior hole mobility compared to that of **BTF4**. To understand the charge transfer behaviors of these dopant-free HTMs, the electrochemical impedance spectroscopy (EIS) of fabricated cells was further measured at open circuit with a frequency ranging from 1 Hz to 1 MHz, and the Nyquist plots are shown in Fig. S14.† Similar to hole mobility, the obtained recombination resistance (*R*_rec_) also shows an order of *R*_rec_(**BTF4**) > *R*_rec_(**BTF3**) > *R*_rec_(**BTF2**) ∼ *R*_rec_(spiro-OMeTAD) > *R*_rec_(**BTF1**). This therefore suggests that **BTF3** and **BTF4** can efficiently reduce the recombination process with higher recombination resistance to produce higher FFs.

**Table 2:** Device parameters of (FAPbI_3_)_0.85_(MAPbBr_3_)_0.15_-based dopant-free PVSCs using **BTF1–4**, spiro-OMeTAD and PEDOT:PSS as HTMs

HTMs	Device	*V*_oc_ (V)	*J*_sc_ (mA cm^–2^)	FF (%)	PCE (%)
**BTF1**	n–i–p	0.91(0.90 ± 0.01)	17.5(16.2 ± 1.3)	62.6(60.7 ± 1.8)	9.97(8.84 ± 1.12)
**BTF2**	n–i–p	0.85(0.84 ± 0.02)	19.5(18.0 ± 1.4)	63.1(60.7 ± 2.1)	10.45(9.16 ± 1.02)
**BTF3**	n–i–p	1.08(1.07 ± 0.02)	20.4(19.2 ± 1.1)	74.3(72.4 ± 1.8)	16.34(15.28 ± 1.13)
**BTF4**	n–i–p	1.06(1.05 ± 0.01)	22.5(21.5 ± 1.1)	75.6(73.7 ± 1.9)	18.03(16.97 ± 1.05)
Spiro-OMeTAD	n–i–p	1.02(1.01 ± 0.01)	14.3(13.0 ± 1.3)	64.0(62.4 ± 1.5)	9.33(8.21 ± 1.09)
**BTF1**	p–i–n	0.96(0.95 ± 0.01)	16.1(14.9 ± 1.2)	72.4(70.7 ± 1.5)	11.19(10.02 ± 0.98)
**BTF2**	p–i–n	0.91(0.90 ± 0.02)	17.7(16.5 ± 1.1)	74.3(72.7 ± 1.6)	11.96(10.51 ± 1.02)
**BTF3**	p–i–n	1.03(1.02 ± 0.01)	19.3(18.3 ± 1.1)	75.9(74.1 ± 1.7)	15.09(14.14 ± 0.96)
**BTF4**	p–i–n	1.03(1.01 ± 0.02)	21.5(20.5 ± 1.0)	76.8(75.3 ± 1.5)	17.01(16.14 ± 0.85)
PEDOT:PSS	p–i–n	0.96(0.94 ± 0.01)	22.4(21.5 ± 1.0)	76.4(74.7 ± 1.6)	16.42(15.48 ± 0.94)

Doped conventional devices were also fabricated using Li–TFSI as the dopant with an additive of 4-*tert*-butyl pyridine (TBP). As shown in Fig. S16,† the device PCEs of doped **BTF1–2** can be enhanced by 40% due to an effective improvement on both *V*_oc_ and FF. Nonetheless, such enhancement is still poor in comparison with that of doped spiro-OMeTAD (18.8%). A slight enhancement on PCEs was observed for the doped **BTF3–4** based devices, while the highest PCE of 18.94% was achieved for **BTF4**. These results, on the other hand, suggest that our dicyano-fluoranthene-cored molecules can serve as highly efficient HTMs without needing dopants.

Today, most HTMs used in inverted p–i–n devices are polymer HTMs such as poly(3,4-ethylenedioxythiophene) polystyrenesulfonate (PEDOT:PSS) and polytriarylamine (PTAA).^46^–^49^ A doping process is also required for PTAA to improve device performance.^50^ PEDOT:PSS belongs to a class of self-doped polymers, but always suffers a large potential loss and inherent acidity-induced stability problem.^51^,^52^ Therefore, it is also important to develop dopant-free HTMs suitable for inverted PVSCs.^53^,^54^ However, few molecular HTMs have been reported to be applied in both conventional and inverted planar PVSCs. To this end, inverted PVSCs with a configuration of ITO/HTL/mixed perovskite/PCBM/LiF/Ag were also tested using **BTF1–4** as the dopant-free HTMs, while a PEDOT:PSS-based control device was fabricated for comparison. The *J*–*V* curves of the best-performing devices are shown in Fig. 3b. From **BTF1** to **BTF4**, the resulting PCEs of the inverted devices increase gradually (Table 2), a similar trend to that observed in conventional devices. The champion PCE from **BTF4**-based inverted PVSCs is 17.01%, higher than that of the PEDOT:PSS-based control device (16.42%).

The stabilized PCE and photocurrent of the champion devices of **BTF4** near the maximum power point (Fig. 3c and S18b†) were tested to evaluate the efficacy of the *J*–*V* curves. The results clearly show the high reliability of our *J*–*V* curve with an absence of current hysteresis, when combined with those *J*–*V* curves with forward and reverse scanning directions at different scan rates (Fig. S12b and S18c†). We also measured the incident photon-to-electron conversion efficiency (IPCE) spectra of all champion conventional and inverted devices based on dopant-free **BTF1–4** (Fig. S15 and S20†), wherein the integrated *J*_sc_ values are consistent with those obtained from the experimental *J*–*V* measurements (Table 2).

To check if removing the doping process can improve the device stability of PVSCs over doped spiro-OMeTAD, we compared the environmental stability of PVSCs derived from dopant-free **BTF4** and doped spiro-OMeTAD without encapsulation. The PCE decay curves are presented in Fig. 3d, wherein it is easily seen that the performance of both devices decreased quickly at the beginning when being stored in ambient air under a relative humidity of 40–50%. However, the dopant-free **BTF4** based device can still retain over 50% of its original PCE after being stored for 30 days, while the PCE of the doped spiro-OMeTAD based device almost vanishes. Moreover, **BTF4**-based inverted devices also showed an enhanced stability and can retain 55% of their original PCE after being stored in air for 7 days, while 90% of the PCE of the PEDOT:PSS-based control device was lost (Fig. S18e†). Thus, our designed fluoranthene-cored dopant-free HTMs not only deliver a high PCE comparable to doped spiro-OMeTAD, but also show an enhanced device stability, suggesting that they are very promising material candidates towards efficient PVSCs.

## Conclusions

In summary, 2,3-dicyano-fluoranthene was first prepared through a new and facile synthetic method based on a Diels–Alder reaction. We found that fluoranthene could be an ideal building block for designing D–A type dopant-free HTMs with low synthetic cost and compatible energy levels with perovskites through rational molecular design. The detailed crystal structure analysis indicates that the resulting molecules with dicyano-substituted fluoranthene as the core present highly ordered and strong molecular packing in solid states, and in particular, **BTF4** forms a quasi-three-dimensional herringbone assembly, leading to a much higher hole mobility (up to 10^–4^ cm^2^ V^–1^ s^–1^) than that of spiro-OMeTAD. Encouragingly, our designed molecules can be applied in planar PVSCs as efficient dopant-free HTMs yielding high device performance, including efficiency and stability. For **BTF4**, impressive PCEs of 18.03% and 17.01% have been achieved for conventional and inverted cells respectively. Therefore, our work not only develops a general material design strategy to achieve efficient dopant-free HTMs for PVSCs, but also provides a new synthetic method to obtain functionalized fluoranthenes.

## Conflicts of interest

There are no conflicts to declare.

## Supplementary Material

Supplementary information

Crystal structure data
