# Supplementary material for: Fluoranthene-based dopant-free hole transporting materials for efficient perovskite solar cells
Source: Chem Sci. 2018 Feb 2;9(10):2698–704. doi: 10.1039/c7sc05484j (PMC5914136; doi:10.1039/c7sc05484j)
Supplement: Supplementary file 1 [file SC-009-C7SC05484J-s001.pdf]

Electronic Supplementary Material (ESI) for Chemical Science.
This journal is © The Royal Society of Chemistry 2018

Supporting Information

Fluoranthene-based Dopant-free Hole Transporting Materials for Efficient Perovskite Solar Cells

Xianglang Sun,[a†] Qifan Xue,[c†] Zonglong Zhu,*[b] Qi Xiao,[a] Kui Jiang,[b] Hin-Lap Yip,[c] He Yan*[b] and Zhong'an Li*[a]

[a] Key Laboratory for Material Chemistry of Energy Conversion and Storage, Ministry of Education, School of Chemistry and Chemical Engineering, Huazhong University of Science and Technology, 430074, Wuhan, China

[b] Department of Chemistry, Energy Institute and Hong Kong Branch of Chinese National Engineering Research Center for Tissue Restoration and Reconstruction, The Hong Kong University of Science and Technology, Clear Water Bay, Kowloon, Hong Kong

c Institute of Polymer Optoelectronic Materials and Devices, State Key Laboratory of Luminescent Materials and Devices, South China University of Technology, Guangzhou, P. R. China

† These two authors contributed this work equally.

* Corresponding Author: lizha@hust.edu.cn; zzhuab@connect.ust.hk; hyan@ust.hk.

Table of Contents

S.1	Synthetic details and characterization data
S.2	X-ray data and crystal structure
S.3	Solution and emission spectra
S.4	TGA and DSC curves
S.5	Characterization data of conventional n-i-p devices
S.6	Characterization data of inverted p-i-n devices
S.7	^{1}H NMR, ^{13}C NMR and HR-Mass Spectra
S.8	Reference

S.1 Synthetic Details and Characterization Data

Instrumentations.

^{1}H NMR and ^{13}C NMR spectra were measured using a Bruker 400 and 600 MHz instrument spectrometers. High-resolution mass spectrometry was performed by using Bruker Daltonics instrument, SolariX 7.0T. Liquid chromatography mass spectrometry was performed using an Agilent 1100 LC/MSD Trap instrument. Cyclic voltammetry was measured on a CHI6001 electrochemical analyzer (CH Instruments, Inc., China) using a conventional three-electrode cell with Pt metal as the working electrode, Pt gauze as the counter-electrode, and Ag/Ag^+ as the reference electrode at a scan rate of 50 mV/s. Thermal transition was measured on a NETZSCH STA 449 F3 Jupiter with a heating rate of 10 °C min^{-1}. UV−vis−IR absorption spectra were measured using a SHIMADZU UV-3600 UV-VIS-NIR spectrophotometer. The photoluminescent spectra were measured on a SHIMADZU RF-5301PC Spectrofluorophotometer.

Materials.

Both toluene and tetrahydrofuran (THF) was dried and distilled from Sodium under an atmosphere of dry nitrogen. Most of reagents were purchased from Adamas (Titan Scientific, Shanghai), except TCNE (Aldrich), $Pd_2(dba)_3$ (Fluorochem), $Pd(dba)_2$(Frontier), CH_3Li (J&K Seal). 9-methylene-9H-fluorene was synthesized according to the previous literature.[1]

Device Fabrication.

Indium tin oxide (ITO)-coated glass substrates were cleaned with isopropanol, acetone, distilled water sequentially, and treated with UV ozone for 30 min. For n-i-p conventional perovskite solar cell, the colloidal SnO_2 nanocrystals was spin-coated onto ITO substrate at at 4000 r.p.m for 60 s and thermal anealling at 150 °C for 30 min. The C_{60} electron transport layer (3 mg/mL in chlorobenzene) was deposited by spin coating at 3000 r.p.m for 30 s and 4000 r.p.m for 5 s. The perovskite precursor solution was then spin-coated onto the above prepared films at 5000 rpm for 30 s. During the last 15 s of the second spin-coating step, the substrate was treated with Chlorobenzene drop-casting (0.1 mL). The as spun films were annealed at 100 °C for ~45 min. The hole transport materials **BTF1-4** (8 mg/mL in chloroform) or typical spiro-OMeTAD (15 mg/ml in chlorobenzene) were spin-coated on top of the

perovskite layer at 4000 r.p.m for 30 s. The **BTF1-4** based devices were annealing at 100 °C for 5 min. The chemical doping process for spiro-OMeTAD was proceeded by adding additive of 28.8 μL tert-butylpyridine (TBP) and 17.5 μL Li-bis(trifluoromethanesulfonyl) imide (Li-TFSI) in acetonitrile solution (420 mg/mL) in 1 mL solution. A 70 nm thick top Au electrode was evaporated under high vacuum. For p-i-n inverted perovskite solar cell, the hole transport materials **BTF1-4** (8 mg/mL in chloroform) or typical PEDOT:PSS were spin-coated onto ITO substrate at at 3000 r.p.m for 60 s and thermal anealling at 100 °C for 15 min. The perovskite precursor solution was processed as the same procedure mentioned above. Finally, a PCBM layer (15 mg/mL) in chlorobenzene was deposited by spin coating at 2000 r.p.m for 30 s and 4000 r.p.m for 5 s. Finally, A 5 nm LiF following with 100 nm thick top Ag electrode was evaporated under high vacuum.

Device *J-V* characteristics were measured under AM1.5G (100mWcm^{-2}) using a Newport Class A solar simulator (94021A, a Xenon lamp with an AM1.5G filter) in air at room temperature. *J~V* characteristics were recorded using a Keithley 2400 source meter unit. Typical cells have device areas of 5.9 mm^2, which are defined by a metal mask with an aperture aligned with the device area. EQEs were measured using an Enlitech QE-S EQE system equipped with a standard Si diode. Monochromatic light was generated from a Newport 300W lamp source. The SEM was measured on JEOL7100F at an accelerating voltage of 10 kV. For steady-state PL, a 514.5 nm ultrafast laser was used as the excitation light source (Laser sources: Ar ion laser, 50 mW). The hole mobilities were measured using the space charge limited current (SCLC) method, employing a device architecture of ITO/V_2O_5/HTMs/V_2O_5/Al, which is the same as that we used in previously reports. The electrochemical impedance spectroscopy (EIS) was measured at open circuit with a frequency ranging between 1Hz and 1MHz with AC amplitude of 10 mA. The Z-View Analyst software was used to model the Nyquist plots from the impedance measurements.

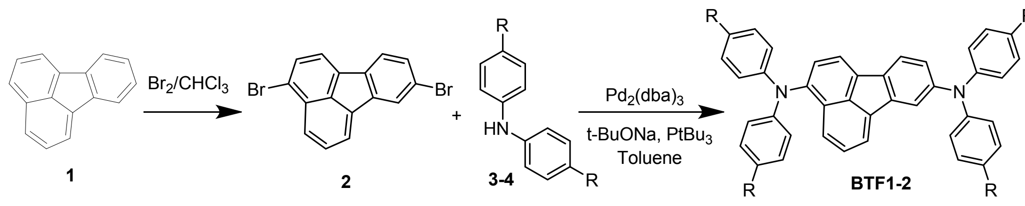

3 and BTF1: R = -Me; **4**, BTF**2**: R = -OMe

Synthesis of 3,8-dibromofluoranthene (2)

This compound was prepared as reported.[2] Fluoranthene (2.02 g, 10.0 mmol) and dichloromethane (DCM, 10 mL) were added to a 100 mL round bottom flask. Afterwards, bromine (3.2 g, 20.0 mmol) in DCM (10 mL) was dropped into the above solution in an ice bath over 20 min. Reaction was then stirred at room temperature for 6 h, and then treated with sodium thiosulfate (10%, 50 mL). The organic layer was then extracted and after removing solvent, the crude product was purified through column chromatography (SiO_2, petroleum ether/DCM, 5/1, V/V) to afford compound **2** (3.40 g, 94.4%) as a yellow solid. ^{1}H NMR (600 MHz, Chloroform-*d*, δ) 8.06 (d, *J* = 8.3 Hz, 1H, ArH), 8.01 (d, *J* = 1.7 Hz, 1H, ArH), 7.94 (d, *J* = 6.9 Hz, 1H, ArH), 7.86 (d, *J* = 7.3 Hz, 1H, ArH), 7.76–7.70 (m, 3H, ArH), 7.52 (dd, *J* = 8.0 Hz, 1.7 Hz, 1H, ArH).

Synthesis of *N*^{3},*N*^{3},*N*^{8},*N*^{8}-tetra-p-tolylfluoranthene-3,8-diamine (BTF1)

A mixture of 3,8-dibromofluoranthene (**2**, 0.72 g, 2.0 mmol), di-p-tolylamine (0.87g, 4.4 mmol), $Pd_2(dba)_3$ (55 mg, 0.06 mmol), NaO*t*Bu (480 mg, 5.0 mmol), P*t*Bu_3 (0.225 Ml, 0.09 mmol) and dry toluene (10 mL) was heated to reflux under N_2 for 24 h. After cooling to room temperature, the mixture was extracted with DCM. The combined organic layer was dried by anhydrous Na_2SO_4 and concentrated, and then was purified through column chromatography (SiO_2, petroleum ether/DCM, 10/1, V/V) to afford **BTF1** (0.78 g, 65.8%) as an orange solid. ^{1}H NMR (400 MHz, Chloroform-*d*, δ): 7.72 (m, 3H, ArH), 7.63 (m, 2H, ArH), 7.35 (t, *J* = 8.0 Hz, 1H, ArH), 6.90-7.15 (br, m, 18H, ArH), 2.36 (s, 6H, $-CH_3$), 2.32 (s, 6H, $-CH_3$). ^{13}C NMR (101 MHz, Chloroform-*d*, δ): 147.60, 146.82, 145.54, 144.25, 140.79, 137.04, 134.63, 133.70, 133.13, 132.17, 131.44, 129.85, 129.69, 127.70, 127.23, 126.73, 124.63, 124.24, 122.90, 122.88, 121.59, 120.08, 117.11, 20.80, 20.73. HRMS (ESI): M^+ = 592.2889 (calcd for $C_{44}H_{36}N_2$, 592.2878).

Synthesis of *N*^{3},*N*^{3},*N*^{8},*N*^{8}-tetrakis(4-methoxyphenyl)fluoranthene-3,8-diamine (BTF2)

A mixture of 3,8-dibromofluoranthene (**2**, 0.72 g, 2.0 mmol), bis(4-methoxyphenyl)amine (1.00 g, 4.4 mmol), $Pd_2(dba)_3$ (55 mg, 0.06 mmol), NaO*t*Bu (0.48 g, 5.0 mmol), P*t*Bu_3 (0.225 mL, 0.09 mmol) and dry toluene (10 mL) was heated to reflux under N_2 for 24 h. After cooling to room temperature, the mixture was extracted with DCM. The combine organic layer was dried by anhydrous Na_2SO_4 and concentrated, and then was purified through column chromatography (SiO_2, petroleum ether/DCM, 3/1, V/V) to afford **BTF2** (0.87 g, 66.2%) as a red solid. ^1H NMR (400 MHz, Chloroform-*d*, δ): 7.73–7.54 (m, 4H, ArH), 7.47 (m, 1H, ArH), 7.30 (t, J = 8.0 Hz, 1H, ArH), 7.10 (m, 5H, ArH), 6.99 (d, J = 8.5 Hz, 4H, ArH), 6.92 (d, J = 8.2 Hz, 1H, ArH), 6.84 (d, J = 8.6 Hz, 4H, ArH), 6.77 (d, J = 8.6 Hz, 4H, ArH), 3.81 (s, 6H, $-OCH_3$), 3.77 (s, 6H, $-OCH_3$). ^{13}C NMR (101 MHz, Chloroform-*d*, δ): 155.56, 154.94, 148.06, 144.65, 143.15, 141.37, 140.66, 137.09, 134.65, 133.16, 132.20, 127.23, 126.96, 126.18, 126.08, 125.48, 124.69, 124.62, 124.46, 121.39, 121.00, 119.88, 119.81, 115.27, 114.65, 114.50, 55.48, 55.45. HRMS (ESI): M^+ = 656.2694 (calcd for $C_{44}H_{36}N_2O_4$, 656.2675).

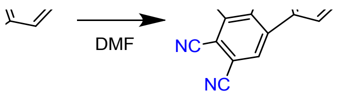

Synthesis of 2,3-dicyano-fluoranthene

9-methylene-9H-fluorene (200 mg, 1.1 mmol) was dissolved in 5 mL of DMF (5 mL) and then TCNE (287 mg, 2.2 mmol) was added. The mixture was heated to 160 °C for 48 h. After the mixture cooled, 20 mL of water was added, and the precipitated yellow solid was collected by filtration and washed with 30 mL of water. The crude product was purified through column chromatography (SiO_2, petroleum ether/DCM, 1:1, V/V) to afford 2,3-dicyano-fluoranthene (10 mg, 3.5%) as a pale yellow solid. ^1H NMR (600 MHz, Chloroform-*d*, δ): 8.14 – 8.04 (m, 3H, ArH), 7.92 (d, J = 7.5 Hz, 2H, ArH), 7.87 (t, J = 6.0 Hz, 1H, ArH), 7.54 (t, J = 6.0 Hz, 1H, ArH), 7.49 (t, J = 6.0 Hz, 1H, ArH). ^{13}C NMR (101 MHz, Chloroform-*d*, δ): 141.47, 138.75, 136.95, 135.82, 132.03, 131.22, 129.64, 128.07, 128.03, 123.81, 123.18, 122.28, 121.41, 119.77, 116.06, 115.44, 113.42, 112.62. MS (APCI): M^+ = 252.0 (calcd for $C_{18}H_8N_2$, 252.0).

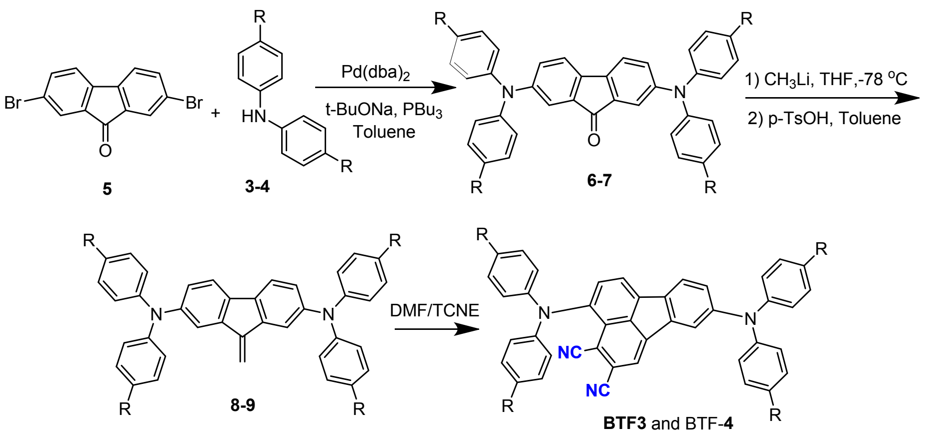

Synthesis of 2,7-bis(di-p-tolylamino)-9H-fluoren-9-one (6)

This compound was prepared as reported.[3] A mixture of 2,7-dibromo-9-fluorenone (**5**, 3.38 g, 10.0 mmol), di-p-tolylamine (4.33 g, 22.0 mmol), Pd(dba)$_2$ (0.37 g, 0.4 mmol), NaO*t*Bu (2.4 g, 25.0 mmol), P*t*Bu$_3$ (1.5 mL, 0.6 mmol) and dry toluene (40 mL) was heated to reflux under N_2 for 24 h. After cooling to room temperature, the mixture was extracted with DCM. The combine organic layer was dried by anhydrous Na_2SO_4 and concentrated, and then was purified through column chromatography (SiO_2, petroleum ether/DCM, 3/1, V/V) to afford **6** (4.95 g, 86.8%) as a dark red solid. ^{1}H NMR (400 MHz, Chloroform-*d*, δ): 7.25 (d, *J* = 2.2 Hz, 2H, ArH), 7.17 (d, *J* = 8.1 Hz, 2H, ArH), 7.10-7.02 (m, 10H, ArH), 6.97 (d, *J* = 8.4 Hz, 8H, ArH), 2.31 (s, 12H, -CH_3).

Synthesis of 2,7-bis(bis(4-methoxyphenyl)amino)-9H-fluoren-9-one (7)

A mixture of 2,7-dibromo-9-fluorenone (**5**, 1.69 g, 5.0 mmol), bis(4-methoxyphenyl)amine (2.29 g, 10.0 mmol), Pd(dba)$_2$ (0.18 g, 0.2 mmol), NaO*t*Bu (1.20 g, 25.0 mmol), P*t*Bu$_3$ (0.75 mL, 0.3 mmol) and dry toluene (40 mL) was heated to reflux under N_2 for 24 h. After cooling to room temperature, the mixture was extracted with DCM. The combine organic layer was dried by anhydrous Na_2SO_4 and concentrated, and then was purified through column chromatography (SiO_2, petroleum ether/DCM, 3/1, V/V) to afford comound **7** (2.60 g, 82.3%) as a dark red solid. ^{1}H NMR (400 MHz, Chloroform-*d*, δ): 7.17 (d, *J* = 2.2 Hz, 2H, ArH), 7.13 (d, *J* = 8.2 Hz, 2H, ArH), 7.03 (d, *J* = 8.8 Hz, 8H, ArH), 6.98–6.90 (m, 2H, ArH), 6.83 (d, *J* = 8.9 Hz, 8H, ArH), 3.80 (s, 12H, -OCH_3).

Synthesis of 9-methylene-*N*^{2},*N*^{2},*N*^{7},*N*^{7}-tetra-p-tolyl-9H-fluorene-2,7-diamine (8)

CH_3Li (1.38 mL, 1.6 M in n-Hexane, 2.2 mmol) was added dropwise to a solution of 2,7-bis(di-p-tolylamino)-9H-fluoren-9-one (**6**, 1.14 g, 2.0 mmol) in THF (5 mL) at -78 °C, and the mixture was gradually warm to room temperature and stirred for overnight. Afterwards, the mixture was quenched with water and extracted with DCM. The organic layer was separated, concentrated and purified through a fast silica gel column (SiO_2, petroleum ether/DCM, 1/1, V/V) to afford the intermediate of 2,7-bis(di-p-tolylamino)-9-methyl-9H-fluoren-9-ol, which then was directly dehydrated to afford **8** in 10 mL of dry toluene by adding *p*-toluenesulfonic acid (34.4 mg, 0.2 mmol). Finally, through the purification using column chromatography (SiO_2, petroleum ether/DCM, 5/1, V/V), **8** was obtained as a yellow solid (0.99 g, 95.8%). ^{1}H NMR (400 MHz, Chloroform-*d*, δ): 7.51–7.33 (br, m, 4H, ArH), 7.17–6.88 (br, m, 18H, ArH), 5.80 (s, 2H, -C=CH_2), 2.31 (s, 12H, -CH_3). ^{13}C NMR (101 MHz, Chloroform-*d*, δ): 146.91, 145.55, 143.08, 139.34, 134.66, 131.97, 129.80, 124.79, 123.99, 119.70, 116.59, 108.12, 20.77 .

Synthesis of *N*^{2},*N*^{2},*N*^{7},*N*^{7}-tetrakis(4-methoxyphenyl)-9-methylene-9H-fluorene-2,7-diamine (9)

CH_3Li (1.38 mL, 1.6 M in n-Hexane, 2.2 mmol) was added dropwise to a solution of 2,7-bis(bis(4-methoxyphenyl)amino)-9H-fluoren-9-one (**7**, 1.27g, 2.0 mmol) in THF (5 mL) at -78 °C, and the mixture was gradually warm to room temperature and stirred for overnight. Afterwards, the mixture was quenched with water and extracted with DCM. The organic layer was separated, concentrated and purified through a fast silica gel column (SiO_2, DCM) to afford the intermediate of 2,7-bis(bis(4-methoxyphenyl)amino)-9-methyl-9H-fluoren-9-ol, which then was directly dehydrated to afford **9** in 15 mL of dry toluene by adding *p*-toluenesulfonic acid (34.4 mg, 0.2 mmol). Finally, through the purification using column chromatography (SiO_2, petroleum ether/DCM, 3/1, V/V), compound **9** was obtained as an orange solid (1.01 g, 79.8%). ^{1}H NMR (400 MHz, Chloroform-*d*, δ): 7.35 (br, m, 4H, ArH), 7.03-6.83 (br, m, 18H, ArH), 5.77 (s, 2H, -C=CH_2), 3.80 (s, 12H, -OCH_3). ^{13}C NMR (101 MHz, Chloroform-*d*, δ): 155.46, 147.40, 143.31, 139.24, 133.93, 125.90, 125.87, 123.04, 119.47, 114.80, 114.65, 107.79, 55.51.

Synthesis of 3, 8-bis(di-p-tolylamino)fluoranthene-4,5-dicarbonitrile (BTF3)

Compound **8** (250 mg, 0.44 mmol) was dissolved in 5 mL of DMF and then TCNE (169 mg, 1.32 mmol) was added. The mixture was heated to 160 °C for 48 h under N_2. After the mixture cooled, 20 mL of water was added the precipitated blue solid was collected by filtration and washed with 30 mL of water. The crude product was purified through column chromatography (SiO_2, petroleum ether/DCM, 1/1, V/V) to afford **BTF3** (82 mg, 29.1%) as a blue solid. ^{1}H NMR (400 MHz, Chloroform-*d*, δ): 7.93 (s, 1H, ArH), 7.80 (d, *J* = 7.5 Hz, 1H, ArH), 7.60 (d, *J* = 8.2 Hz, 1H, ArH), 7.51 (d, *J* = 2.2 Hz, 1H, ArH), 7.37 (d, *J* = 7.5 Hz, 1H, ArH), 7.13 (d, *J* = 7.9 Hz, 4H, ArH), 7.10–6.99 (br, m, 9H, ArH), 6.91 (d, *J* = 8.2 Hz, 4H, ArH), 2.36 (s, 6H, $-CH_3$), 2.29 (s, 6H, $-CH_3$). ^{13}C NMR (101 MHz, Chloroform-*d*, δ): 149.23, 146.07, 144.72, 143.33, 142.34, 138.17, 135.62, 135.60, 133.59, 133.36, 132.26, 131.89, 130.22, 129.74, 128.17, 125.13, 123.72, 123.38, 122.79, 122.53, 121.59, 119.21, 116.72, 115.88, 111.31, 20.91, 20.75. HRMS (APCI): $(M+H)^+$ = 643,2902 (calcd for $C_{46}H_{35}N_4^+$, 643.2862).

Synthesis of 3,8-bis(bis(4-methoxyphenyl)amino)fluoranthene-4,5-dicarbonitrile (BTF4)

Compound **9** (250 mg, 0.24 mmol) was dissolved in 5 mL of DMF and then TCNE (90 mg, 0.71 mmol) was added. The mixture was heated to 160 °C under N_2 for 48 h. After the mixture cooled, 20 mL of water was added and the precipitated blue solid was collected by filtration and washed with 30 mL of water. The crude product was purified through column chromatography (SiO_2, petroleum ether/DCM, 1/2, V/V) to afford **BTF4** (72 mg, 43.0%) as a blue solid. ^{1}H NMR (400 MHz, Chloroform-*d*, δ): 7.92 (s, 1H, ArH), 7.76 (d, *J* = 7.6 Hz, 1H, ArH), 7.57 (d, *J* = 8.3 Hz, 1H, ArH), 7.42 (d, *J* = 2.2 Hz, 1H, ArH), 7.31 (d, *J* = 7.4 Hz, 1H, ArH), 7.11 (d, *J* = 8.6 Hz, 4H, ArH), 6.96 (m, 5H, ArH), 6.89 (d, *J* = 8.4 Hz, 4H, ArH), 6.78 (d, *J* = 8.4 Hz, 4H, ArH), 3.83 (s, 6H, $-OCH_3$), 3.77 (s, 6H, $-COH_3$). ^{13}C NMR (101 MHz, Chloroform-*d*, δ): 156.45, 155.39, 149.64, 143.58, 142.43, 142.41, 140.26, 138.09, 135.64, 135.26, 132.63, 130.95, 127.89, 126.97, 124.24, 123.45, 122.43, 121.49, 121.46, 119.00, 116.75, 116.03, 114.98,114.87, 114.49, 55.54, 55.49. HRMS (APCI): $(M+H)^+$ = 707.2631 (calcd for $C_{46}H_{35}N_4O_4^+$, 707.2658).

Table S1. Synthetic cost for the synthesis of **BTF1**.

Chemical	Weight reagent (g/g)	Weight solvent (g/g)	Weight workup (g/g)	Price of Chemical ($/Kg)	Chemical cost $/g product)	Cost per step ($/step)
Fluoranthene (adamas)	0.60			330.00	0.20	
CH_2Cl_2 (adamas)		3.90		1.73	0.00	
Bromine(general source)	0.95			13.55	0.01	
Na_2SO_4(general source)			1.00	3.29	0.00	
CH_2Cl_2(general source)			65.00	0.91	0.06	
Silica gel(general source)			50.00	8.73	0.44	
Petroleum ether(general source)			300.00	1.74	0.52	
						1.23
3,8-dibromofluoranthene (**1**)	0.92					
p,p'-ditolylamine (adamas)	1.12			1477.41	1.65	
$Pd_2(dba)_3$(Fluorochem)	0.07			32198.80	2.25	
NaO*t*Bu(adamas)	0.62			311.75	0.19	
P*t*Bu_3(10% solution, adamas)	0.29			2634.23	0.76	
Toluene(general source)		11.10		6.40	0.07	
CH_2Cl_2(general source)			70.00	1.73	0.12	
Na_2SO_4(general source)			1.50	3.29	0.00	
Silica gel(general source)			50.00	8.73	0.44	
Petroleum ether(general source)			300.00	1.74	0.52	
						**7.13**

Table S2. Synthetic cost for the synthesis of **BTF2**.

Chemical	Weight reagent (g/g)	Weight solvent (g/g)	Weight workup (g/g)	Price of Chemical ($/g)	Chemical cost ($/g product)	Cost per step ($/step)
Fluoranthene (adamas)	0.59			330.00	0.19	
CH_2Cl_2(adamas)		3.51		1.73	0.01	
Bromine (general source)	0.85			13.55	0.01	
Na_2SO_4(general source)			1.0	3.29	0.00	
CH_2Cl_2(general source)			66	0.91	0.06	
Silica gel(general source)			50	8.73	0.43	
Petroleum ether(general source)			130	1.74	0.22	0.92
3,8-dibromofluoranthene (**1**)	0.83					
bis(4-methoxyphenyl)amine (adamas)	1.15			6042.17	6.95	
$Pd_2(dba)_3$(Fluorochem)	0.06			32198.80	1.93	
NaO*t*Bu(adamas)	0.55			311.75	0.17	
P*t*Bu_3(10% solution, adamas)	0.26			2634.23	0.68	
Toluene(general source)		11.0		6.40	0.07	
CH_2Cl_2(general source)			66	0.91	0.06	
Na_2SO_4(general source)			1.5	3.29	0.00	
Silica gel(general source)			50	8.73	0.44	
Petroleum ether(general source)			200	1.74	0.35	**11.41**

Table S3. Synthetic cost for the synthesis of **BTF3**.

**Chemical**	**Weight reagent (g/g)**	**Weight solvent (g/g)**	**Weight workup (g/g)**	**Price of Chemical ($/g)**	**Chemical cost ($/g product)**	**Cost per step ($/step)**
2,7-dibromo-9-fluorenone	0.68			1807.22	1.23	
p,p'-ditolylamine (adamas)	0.88			1477.41	1.30	
$Pd(dba)_2$((Frontier)	0.07			22771.08	1.59	
NaO*t*Bu(adamas)	0.48			311.75	0.15	
P*t*Bu_3(10% solution, adamas)	0.34			2634.23	0.90	
Toluene (general source)		7.20		6.40	0.04	
CH_2Cl_2 (general source)			120	0.91	0.11	
Na_2SO_4 (general source)			0.5	3.29	0.00	
Silica gel (general source)			50	8.73	0.44	
Petroleum ether (general source)			120	1.74	0.21	
						5.97
CH_3Li()	0.05			963.41	0.05	
2,7-bis(di-p-tolylamino)-9H-fluoren-9-one	1.16					
THF(adamas)		5.08		6.76	0.03	
DCM (general source)			140	0.91	0.13	
Na_2SO_4(general source)			0.7	3.29	0.00	
Silica gel(general source)			50	8.73	0.44	
Petroleum ether(general source)			120	1.74	0.21	
p-toluenesulfonic acid(general source)	0.04			33.89	0.00	
						7.79
9-methylene-*N*2,*N*2,*N*7,*N*7-tetra-p-tolyl-9H-fluorene-2,7-diamine	3.05					
DMF(general source)		57.80		4.53	0.26	
TCNE(Aldrich)	2.06			13638.25	28.09	
Silica gel(general source)			50	8.73	0.44	
Petroleum ether(general source)			260	1.74	0.45	
DCM(general source)			130	0.91	0.12	
						**53.12**

Table S4. Synthetic cost for the synthesis of **BTF4**

**Chemical**	**Weight reagent (g/g)**	**Weight solvent (g/g)**	**Weight workup (g/g)**	**Price of Chemical ($/g)**	**Chemical cost($/g product)**	**Cost per step ($/step)**
2,7-dibromo-9-fluorenone	0.65			1807.22	1.17	
bis(4-methoxyphenyl)amine (adamas)	0.88			6042.17	5.32	
$Pd(dba)_2$(Frontier)	0.07			22771.08	1.59	
NaO*t*Bu(adamas)	0.46			311.75	0.14	
P*t*Bu_3(10% solution, adamas)	0.20			2634.23	0.53	
Toluene(general source)		13.61		6.40	0.09	
CH_2Cl_2(general source)			110	0.91	0.10	
Na_2SO_4(general source)			1	3.29	0.00	
Silica gel(general source)			50	8.73	0.44	
Petroleum ether(general source)			90	1.74	0.16	
						9.54
CH_3Li()	0.05			963.41	0.04	
2,7-bis(di-p-tolylamino)-9H-fluoren-9-one	1.26					
THF(adamas)		4.69		6.76	0.03	
DCM			120	0.91	0.11	
Na_2SO_4(general source)			1.5	3.29	0.00	
Silica gel(general source)			50	8.73	0.44	
Petroleum ether(general source)			80	1.74	0.14	
p-toluenesulfonic acid	0.03			33.89		12.78
*N*2,*N*2,*N*7,*N*7-tetrakis(4-methoxyphenyl)-9-methylene-9H-fluorene-2,7-diamine	3.47					
DMF(general source)		65.84		4.53	0.30	
TCNE(Aldrich)	1.25			13638.25	17.05	
Silica gel(general source)			50	8.73	0.44	
Petroleum ether(general source)			300	1.74	0.52	
DCM(general source)			130	0.91	0.12	
						**62.78**

S.2 X-ray data and crystal structure

Crystallographic data (excluding structure factors) for the structure(s) reported in this paper have been deposited with the Cambridge Crystallographic Data Centre as supplementary publication no. CCDC 1589253 (**BTF1**), CCDC 1589258 (**BTF3**), CCDC 1589261 (**BTF4**). Copies of the data can be obtained free of charge from www.ccdc.cam.ac.uk/conts/retrieving.html .

Table S5. Crystal data and structure refinement for **BTF3**.

Identification code	hkl5	
Empirical formula	C46 H34 N4	
Formula weight	642.77	
Temperature	298(2) K	
Wavelength	0.71073 Å	
Crystal system	Triclinic	
Space group	P-1	
Unit cell dimensions	a = 11.856(7) Å	□= 102.687(8)°.
	b = 12.147(7) Å	□= 111.422(8)°.
	c = 13.446(8) Å	□ = 93.005(9)°.
Volume	1739.7(17) $Å^3$	
Z	2	
Density (calculated)	1.227 Mg/m^3	
Absorption coefficient	0.072 mm^{-1}	
F(000)	676	
Crystal size	0.160 x 0.040 x 0.020 mm^3	
Theta range for data collection	1.683 to 25.000°.	
Index ranges	-14<=h<=14, -14<=k<=14, -15<=l<=15	
Reflections collected	5978	
Independent reflections	5978 [R(int) = 0.0557]	
Completeness to theta = 25.000°	98.6 %	
Absorption correction	None	
Refinement method	Full-matrix least-squares on F^2	
Data / restraints / parameters	5978 / 0 / 457	
Goodness-of-fit on F^2	1.030	
Final R indices [I>2sigma(I)]	R1 = 0.0712, wR2 = 0.1970	
R indices (all data)	R1 = 0.1331, wR2 = 0.2394	
Extinction coefficient	0.010(3)	
Largest diff. peak and hole	0.292 and -0.281 $e.Å^{-3}$	

Table S6. Crystal data and structure refinement for **BTF4**.

Identification code	mo_1705231_0m	
Empirical formula	C46 H34 N4 O4	
Formula weight	706.77	
Temperature	100(2) K	
Wavelength	0.71073 Å	
Crystal system	Monoclinic	
Space group	$P2_1/c$	
Unit cell dimensions	a = 14.1565(18) Å	a= 90°.
	b = 17.917(2) Å	b= 99.826(2)°.
	c = 14.4208(18) Å	g = 90°.
Volume	3604.1(8) $Å^3$	
Z	4	
Density (calculated)	1.303 Mg/m^3	
Absorption coefficient	0.084 mm^{-1}	
F(000)	1480	
Crystal size	0.120 x 0.100 x 0.100 mm^3	
Theta range for data collection	1.829 to 25.497°.	
Index ranges	-17<=h<=17, -21<=k<=21, -17<=l<=15	
Reflections collected	25692	
Independent reflections	6686 [R(int) = 0.0411]	
Completeness to theta = 25.242°	99.7 %	
Refinement method	Full-matrix least-squares on F^2	
Data / restraints / parameters	6686 / 46 / 574	
Goodness-of-fit on F^2	1.035	
Final R indices [I>2sigma(I)]	R1 = 0.0569, wR2 = 0.1487	
R indices (all data)	R1 = 0.0899, wR2 = 0.1833	
Extinction coefficient	n/a	
Largest diff. peak and hole	0.723 and -0.284 e.$Å^{-3}$	

Table S7. Crystal data and structure refinement for **BTF1**.

Identification code	mo_171030a_0m	
Empirical formula	C44 H36 N2	
Formula weight	592.75	
Temperature	100(2) K	
Wavelength	0.71073 Å	
Crystal system	Monoclinic	
Space group	$P2_1/c$	
Unit cell dimensions	a = 8.6947(17) Å	a= 90°.
	b = 17.563(4) Å	b= 97.361(4)°.
	c = 10.737(2) Å	g = 90°.
Volume	1626.1(6) $Å^3$	
Z	2	
Density (calculated)	1.211 Mg/m^3	
Absorption coefficient	0.070 mm^{-1}	
F(000)	628	
Crystal size	0.060 x 0.030 x 0.020 mm^3	
Theta range for data collection	2.237 to 24.996°.	
Index ranges	-10<=h<=8, -20<=k<=20, -12<=l<=12	
Reflections collected	11461	
Independent reflections	2832 [R(int) = 0.1081]	
Completeness to theta = 24.996°	98.7 %	
Absorption correction	None	
Refinement method	Full-matrix least-squares on F^2	
Data / restraints / parameters	2832 / 69 / 259	
Goodness-of-fit on F^2	1.115	
Final R indices [I>2sigma(I)]	R1 = 0.1127, wR2 = 0.3160	
R indices (all data)	R1 = 0.1702, wR2 = 0.3594	
Extinction coefficient	0.009(6)	
Largest diff. peak and hole	1.039 and -0.543 e.$Å^{-3}$	

Note: The intensities of diffraction peaks were not strong, resulting in the values of R1 and wR2 too high. This is mainly Due to the small size and relatively low quality of crystal of **BTF1**.

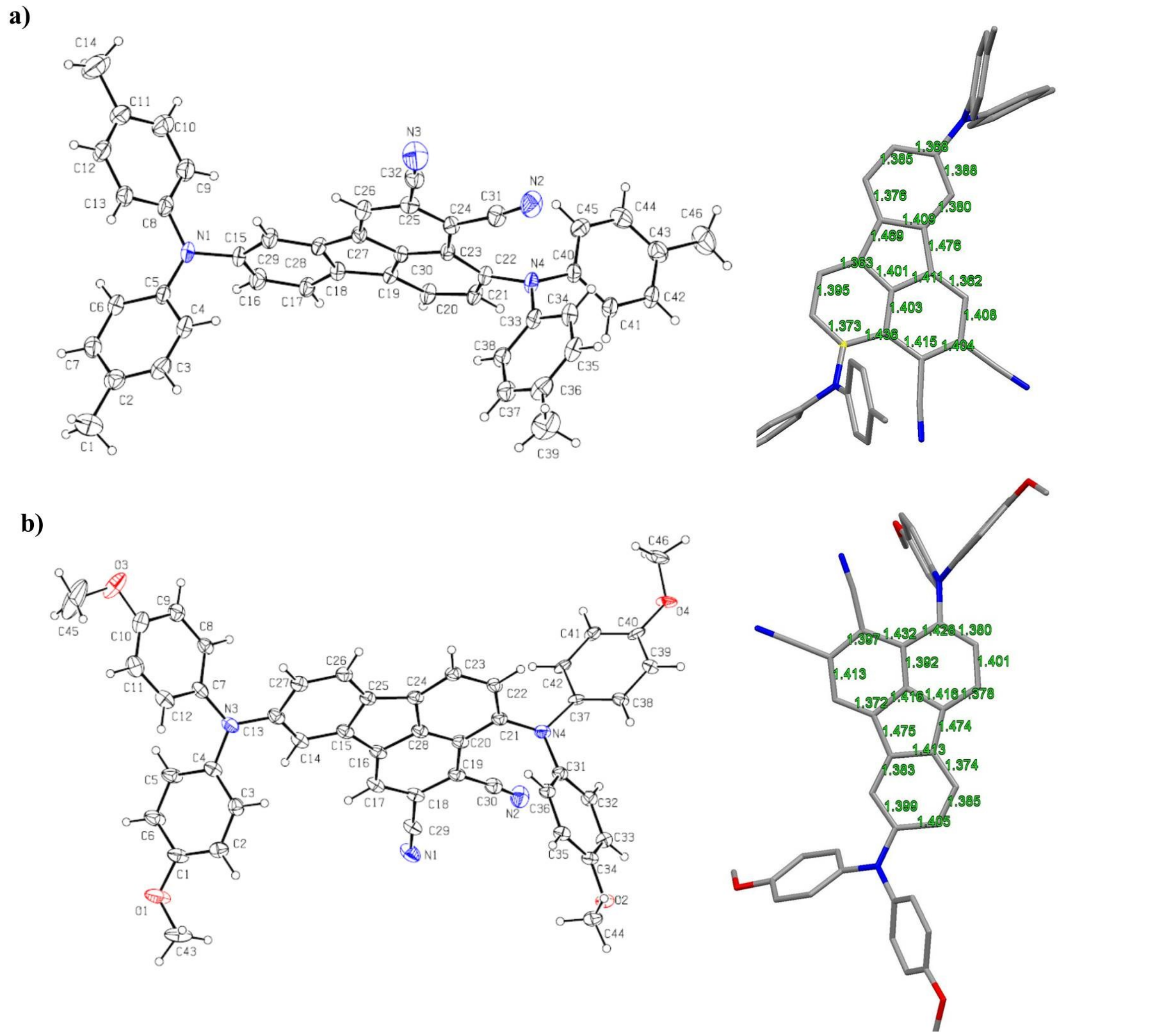

Figure S1. ORTEP structure of **BTF3** (a) and **BTF4** (b), with bond lengths listed in the fluoranthene core.

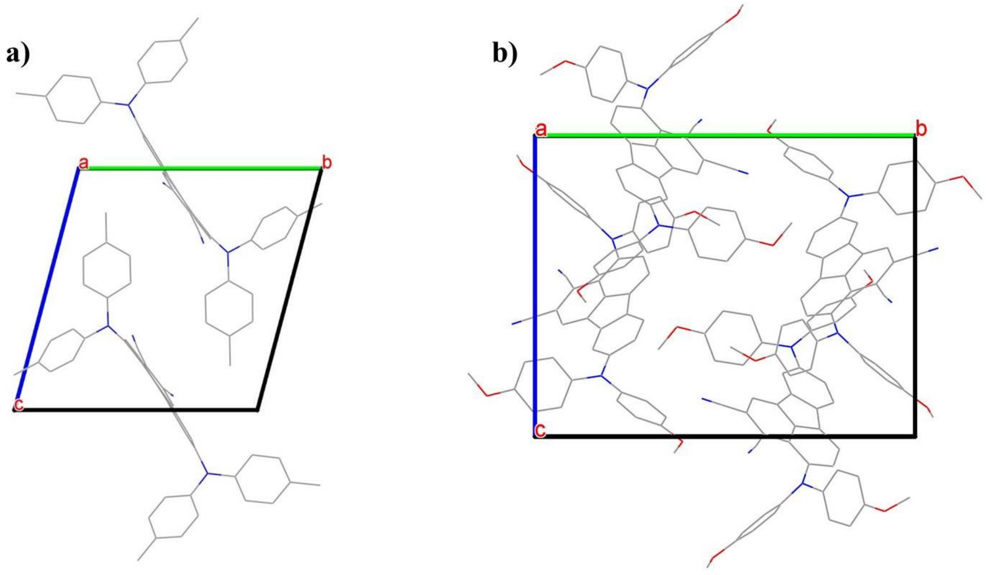

Figure S2. Unit cell of **BTF3 (a)** and **BTF4 (b)**

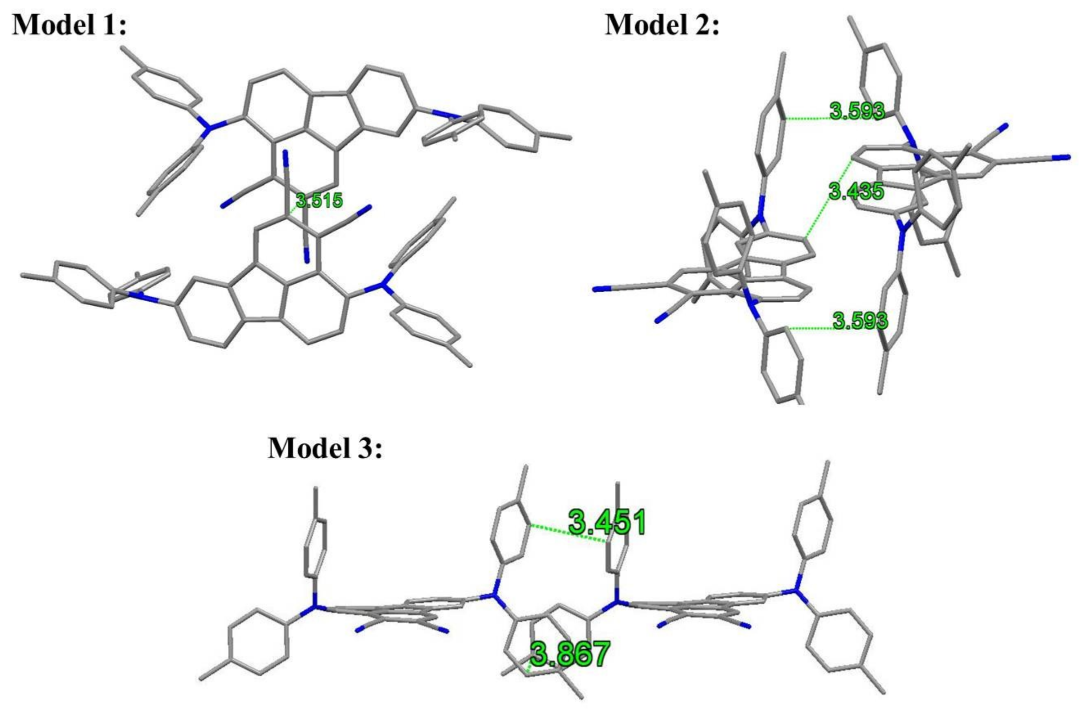

Figure S3. Stack modes of single crystal of **BTF3**.

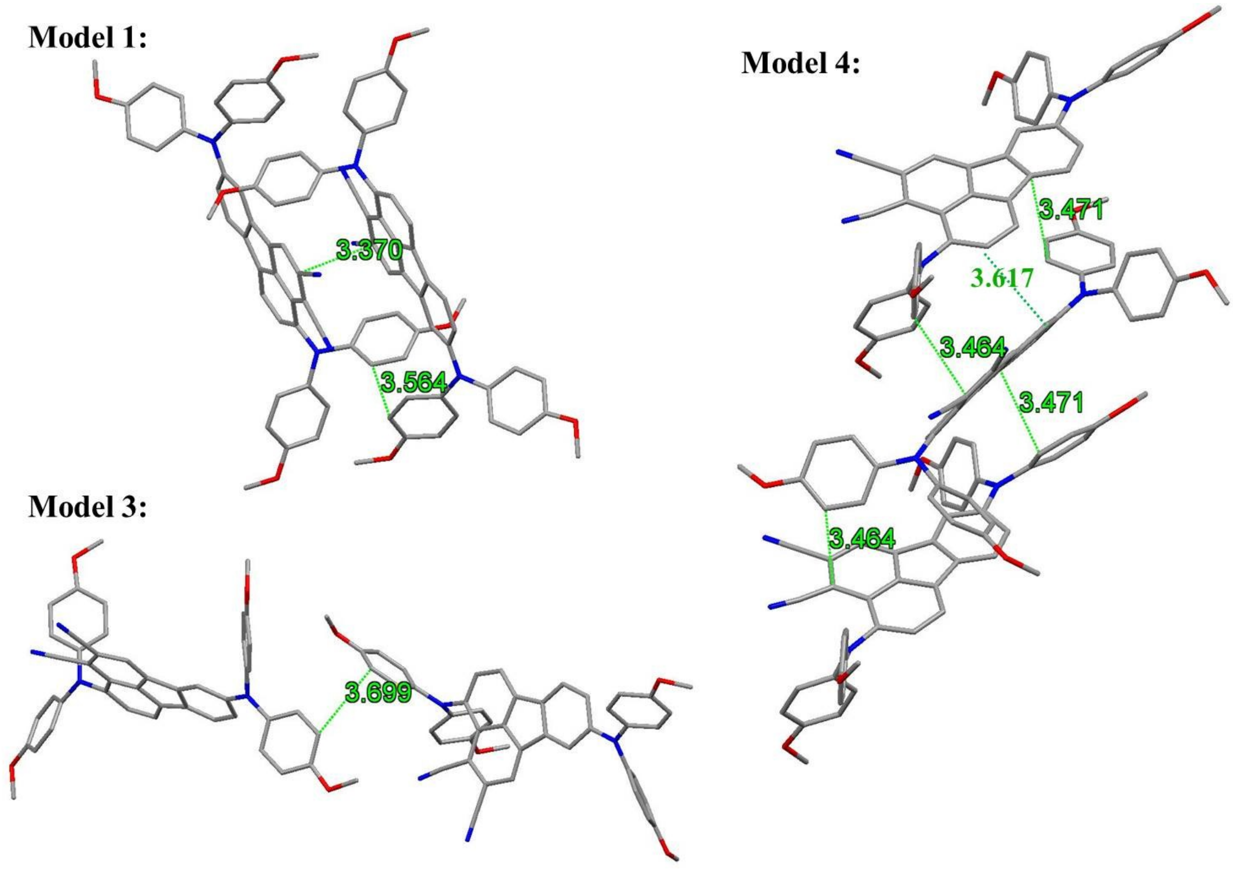

Figure S4. Three stack modes of single crystal of **BTF4**.

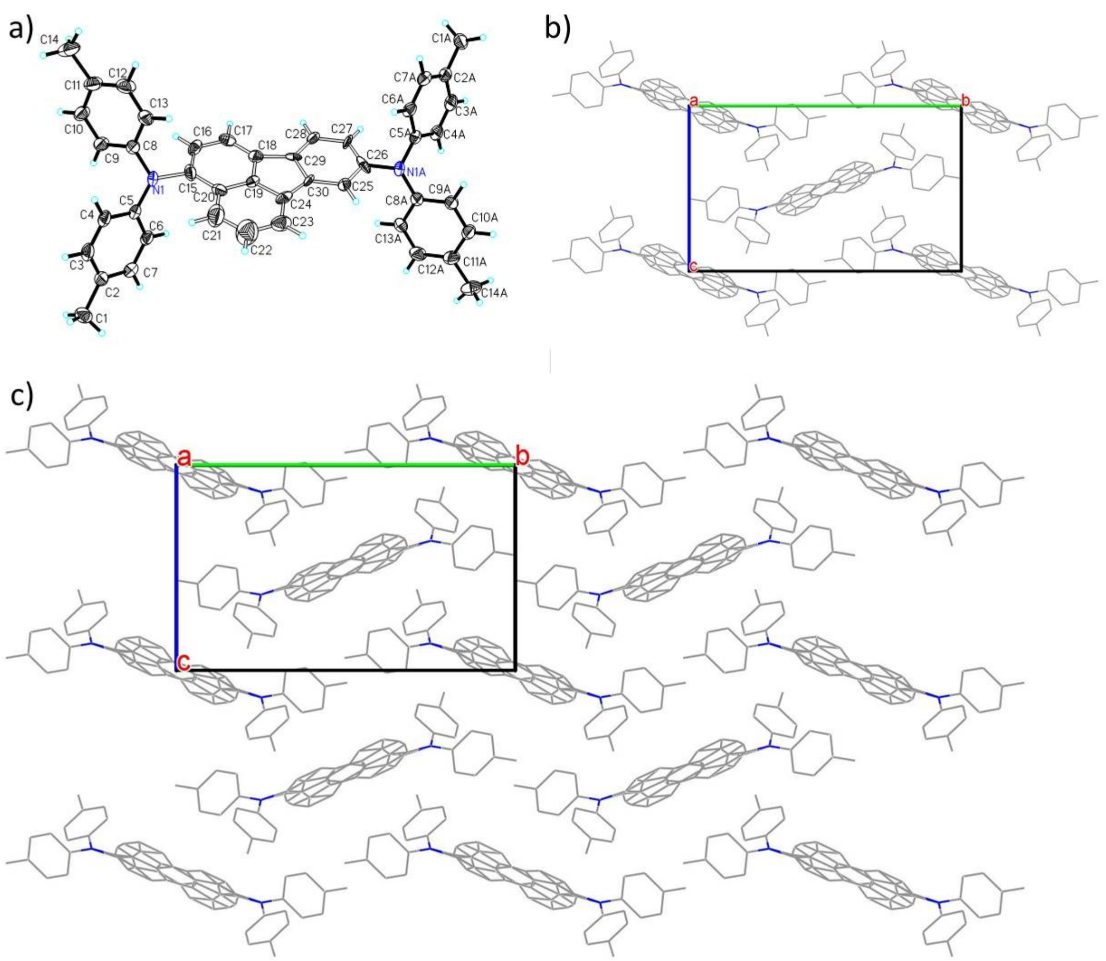

Figure S5. ORTEP structure of **BTF1** (a), Unit cell of **BTF1** (b) and π-stacks of **BTF1** viewed along *a* axis (c). **Note:** The unique mirror structure observed from **BTF1** in the crystal is because the molecules just locate at the center of symmetry (0.5, 1, 1).

S.3. Solution and emission spectra

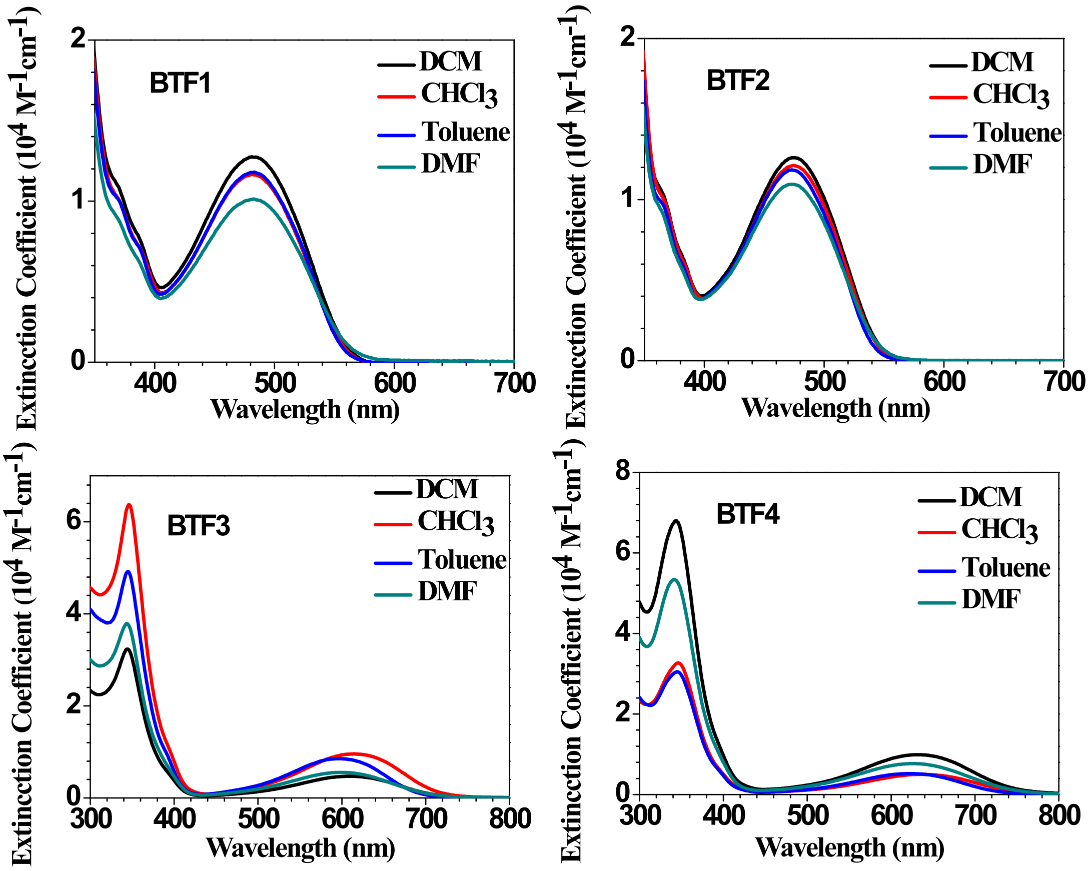

Figure S6. Diluted solution spectra of **BTF1-4** in various organic solvents.

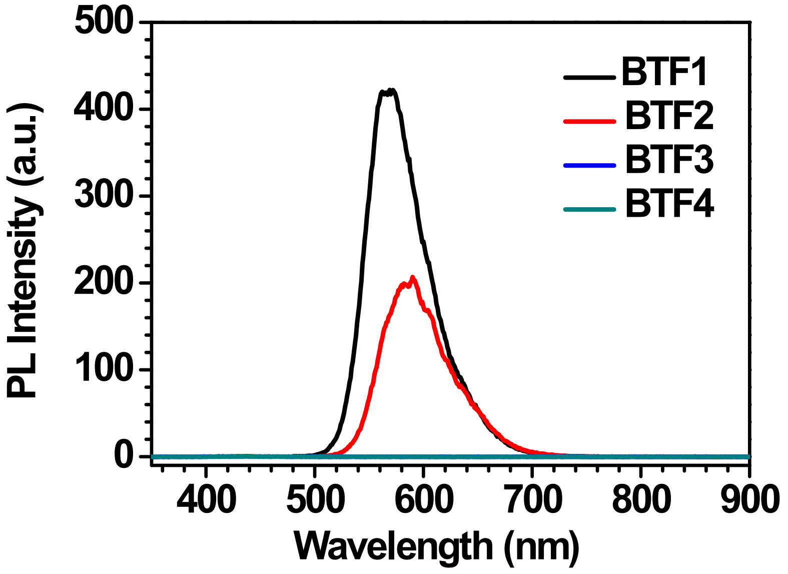

Figure S7. Emission spectra of **BTF1-4** in DCM. Note: **BTF3** and **BTF4** are non-luminescent.

S.4. TGA and DSC curves

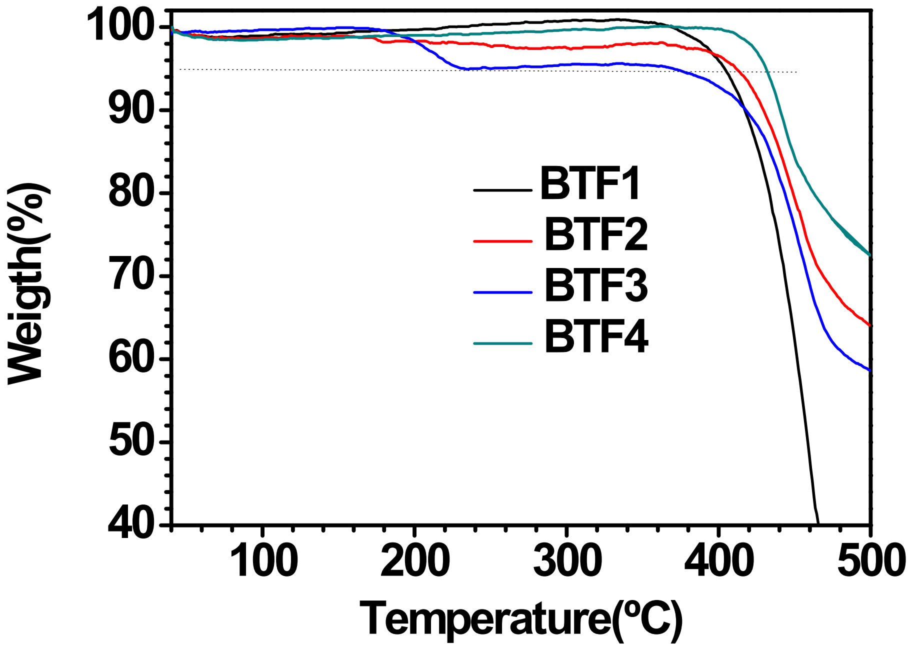

Figure S8. TGA curves of **BTF1-4** under nitrogen with a heating rate of 10 °C/min.

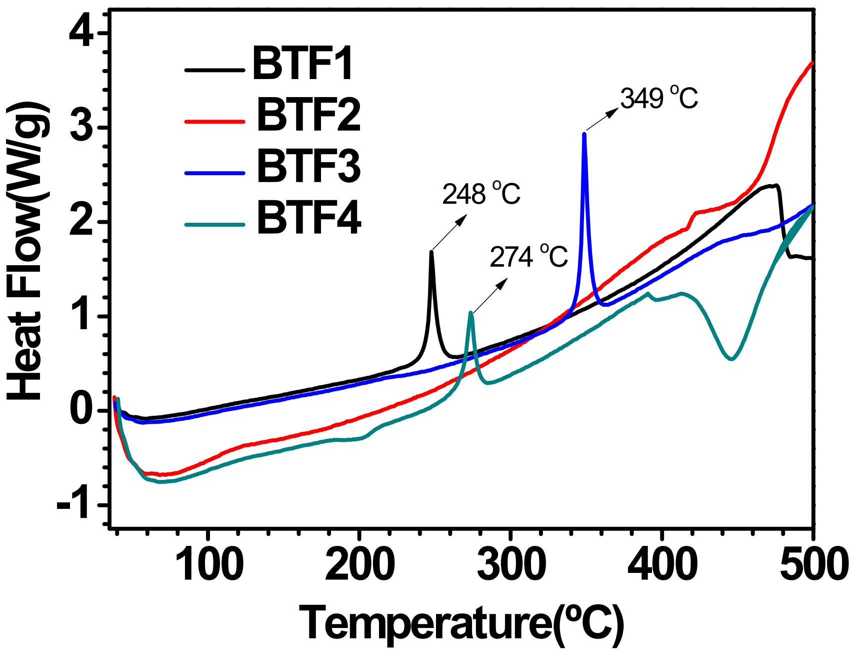

Figure S9. DSC curves of **BTF1-4** under nitrogen with a heating rate of 10 °C/min.

S.5 Characterization data of conventional n-i-p devices

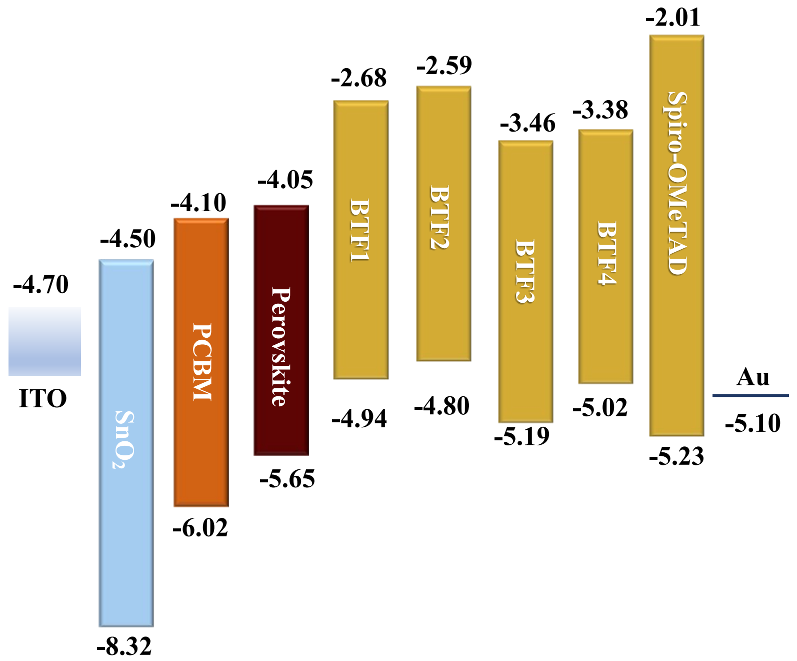

Figure S10. Energy diagram of HTMs in Conventional n-i-p PVSCs.

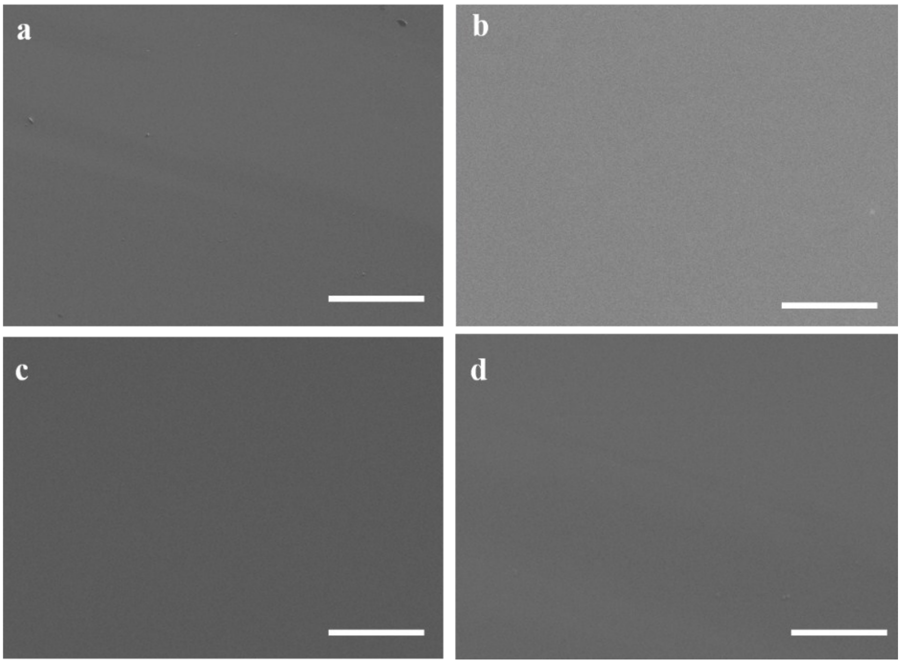

Figure S11. Top view images of scanning electron microscopy (SEM) of **BTF1** (a), **BTF2** (b), **BTF3** (c), **BTF4** (d) films onto perovskites. Note: The scale bar is 1 µm.

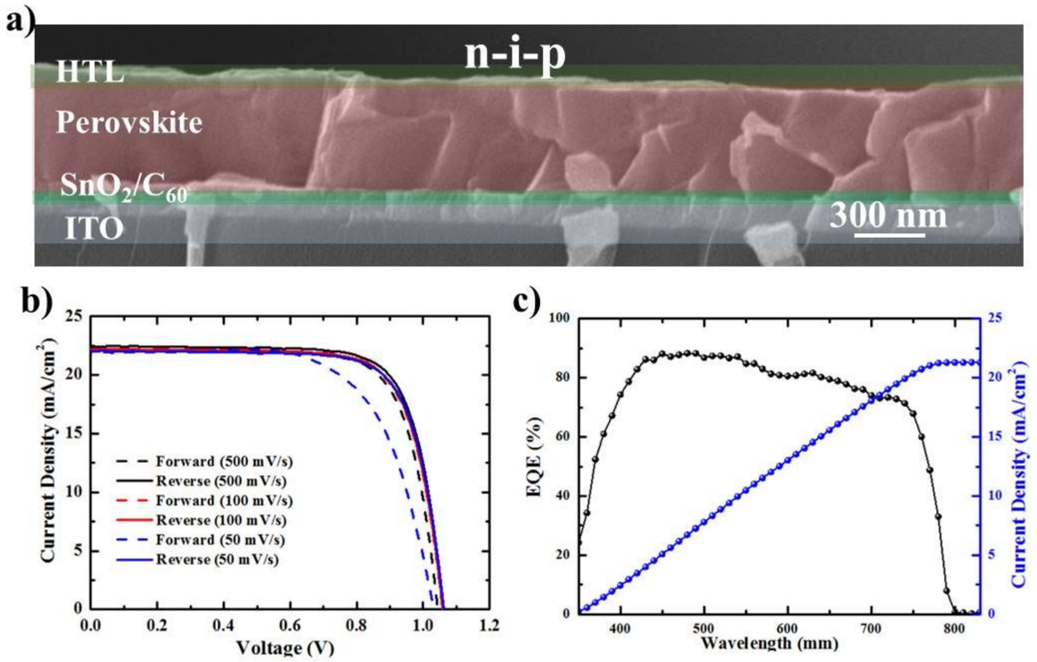

Figure S12. (a) Cross-sectional SEM image of whole conventional n-i-p device based on dopant-free **BTF4**. (b) The photovoltaic performance of the dopant-free **BTF4** -based inverted p-i-n PVSCs measured under forward and reverse scan direction with different scan rate of 50 mV/s, 100 mV/s, and 500 mV/s, respectively. (c) IPCE spectra of **BTF4**-based conventional PVSCs.

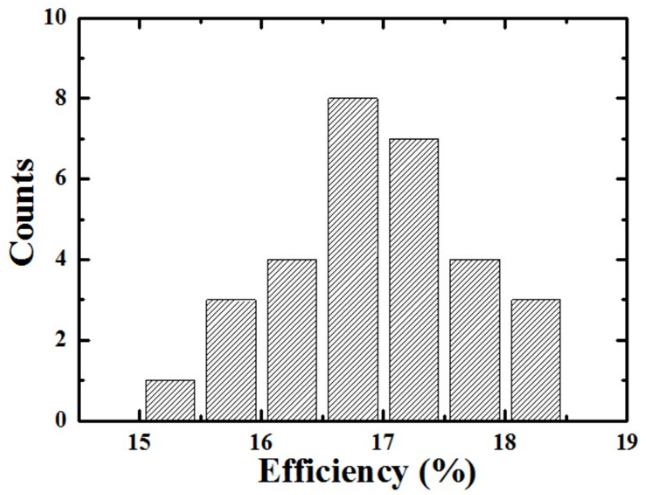

Figure S13. Histogram of efficiency distributions among dopant-free **BTF4** based 30 individual conventional n-i-p cells with a device configuration of ITO/SnO_2/C_{60}/perovskite/**BTF4**/Au.

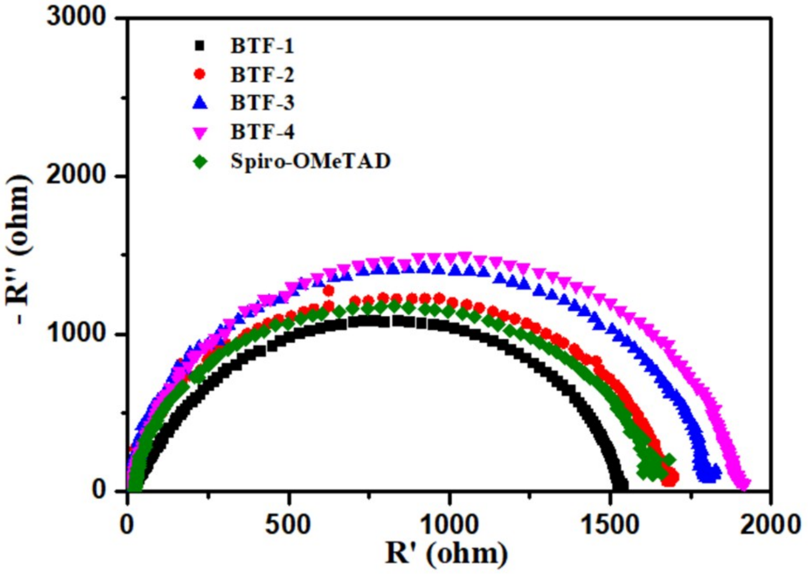

Figure S14. The Nyquist plots of **BTF1-4** based convetional PVSCs under illumination at open circuit status.

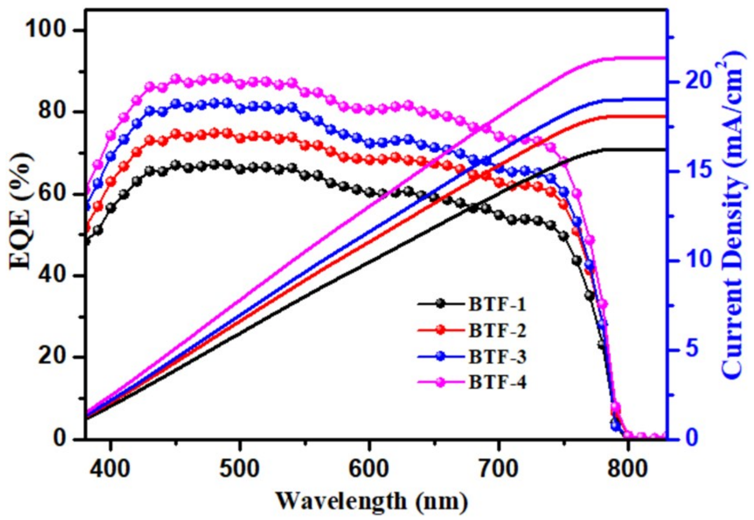

Figure S15. EQE spectra of **BTF1-4** based convetional PVSCs.

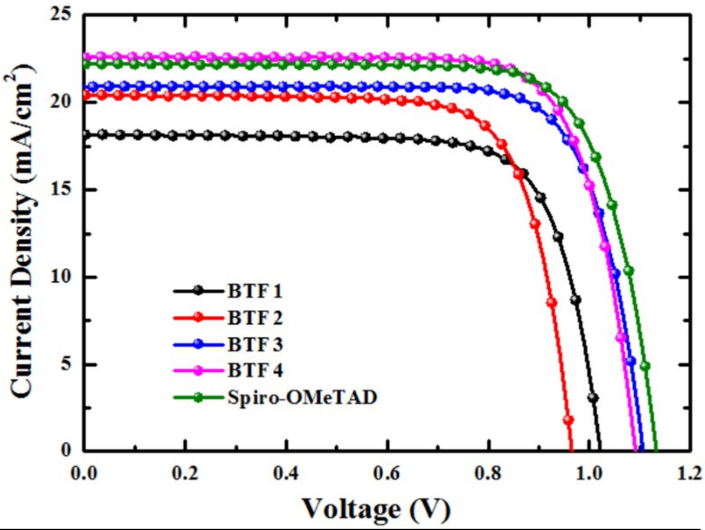

HTMs[a]	V_{oc} (V)	J_{sc} (mA/cm^2)	FF (%)	PCE (%)
doped **BTF1**	1.02	18.2	74.6	13.85
doped **BTF2**	0.96	20.5	74.1	14.85
doped **BTF3**	1.10	20.8	76.5	17.5
doped **BTF4**	1.08	22.6	77.4	18.94
doped Spiro-OMeTAD	1.12	22.1	76.2	18.8

Figure S16. *J*-*V* curves and device parameters of champion conventional n-i-p PVSCs based on the different chemically doped HTMs with structure of ITO/SnO_2/C_{60}/perovskite/doped HTM/Au.

S.6. Characterization data of inverted p-i-n devices

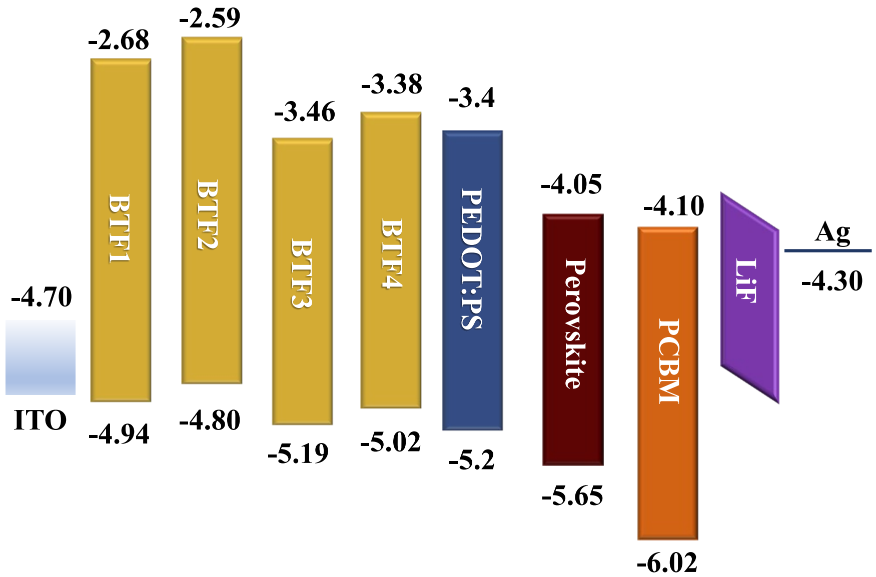

Figure S17. Energy diagram of HTMs in inverted p-i-n PVSCs.

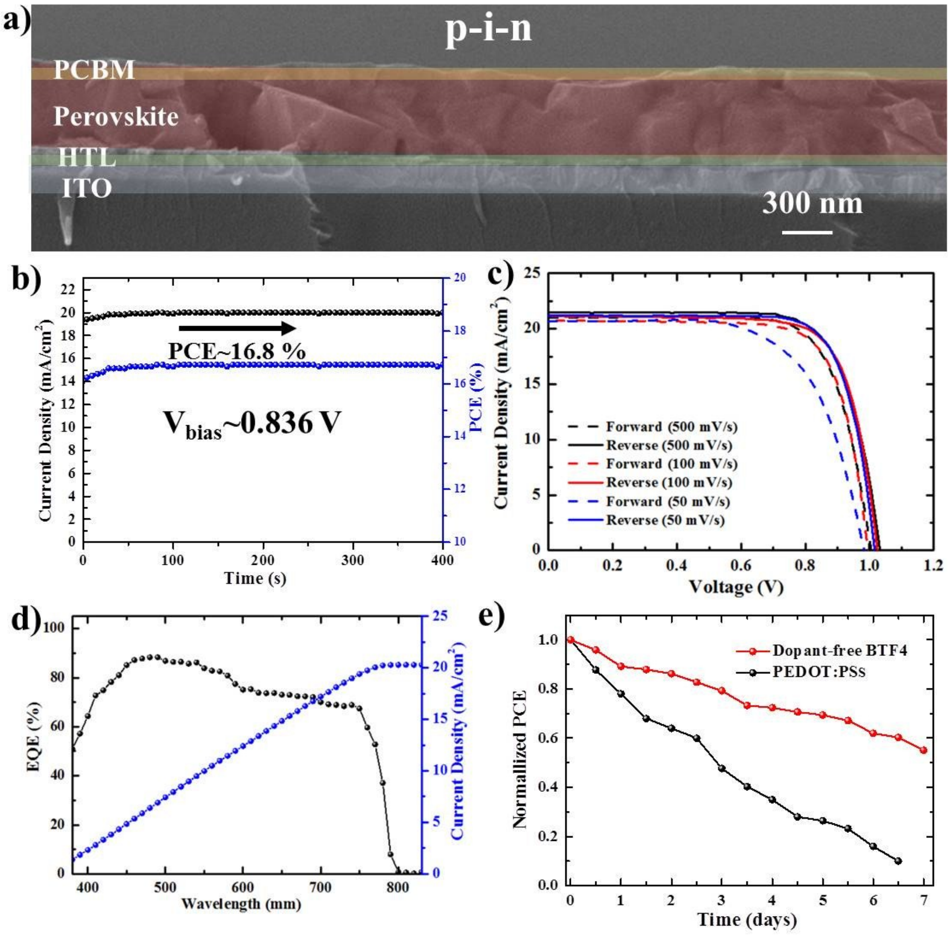

Figure S18. (a) Cross-sectional SEM image of whole inverted device based on dopant-free **BTF4**. (b) Stable output current measured under a constant bias of 0.836 V near the maximum power point. (c) The photovoltaic performance of the dopant-free **BTF4** -based inverted p-i-n PVSCs measured under forward and reverse scan direction with different scan rate of 50 mV/s, 100 mV/s, and 500 mV/s, respectively. (d) IPCE spectra of **BTF4**-based inverted PVSCs. (e) The stability test of the inverted PVSCs in ambient air with a humidity of 40~50% based on dopant-free **BTF4** and PEDOT:PSS as the HTLs.

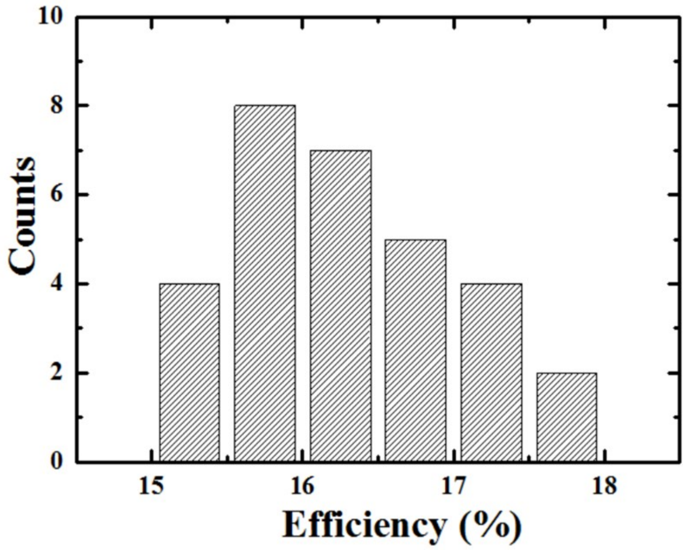

Figure S19. Histogram of efficiency distributions among dopant-free **BTF4** based 30 individual inverted p-i-n cells with a device configuration of ITO/**BTF4**/perovskite/PCBM/LiF.

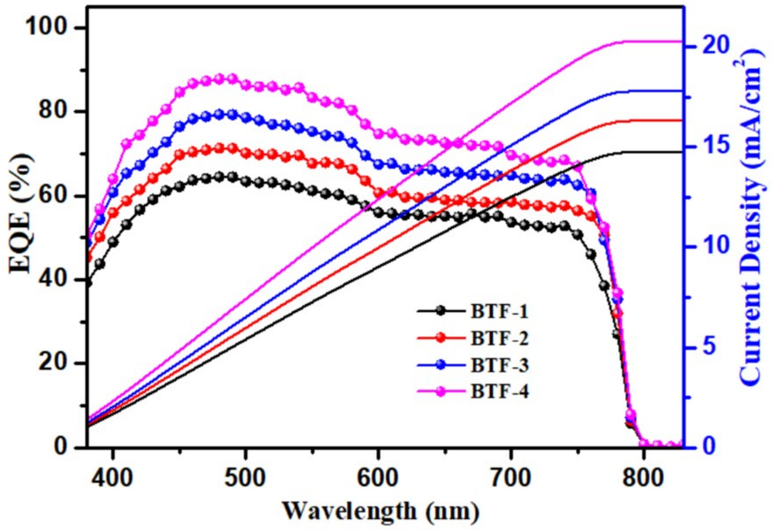

Figure 20. EQE spectra of **BTF1-4** based inverted PVSCs.

S.6. ^{1}H NMR, ^{13}C NMR and HR-Mass Spectra

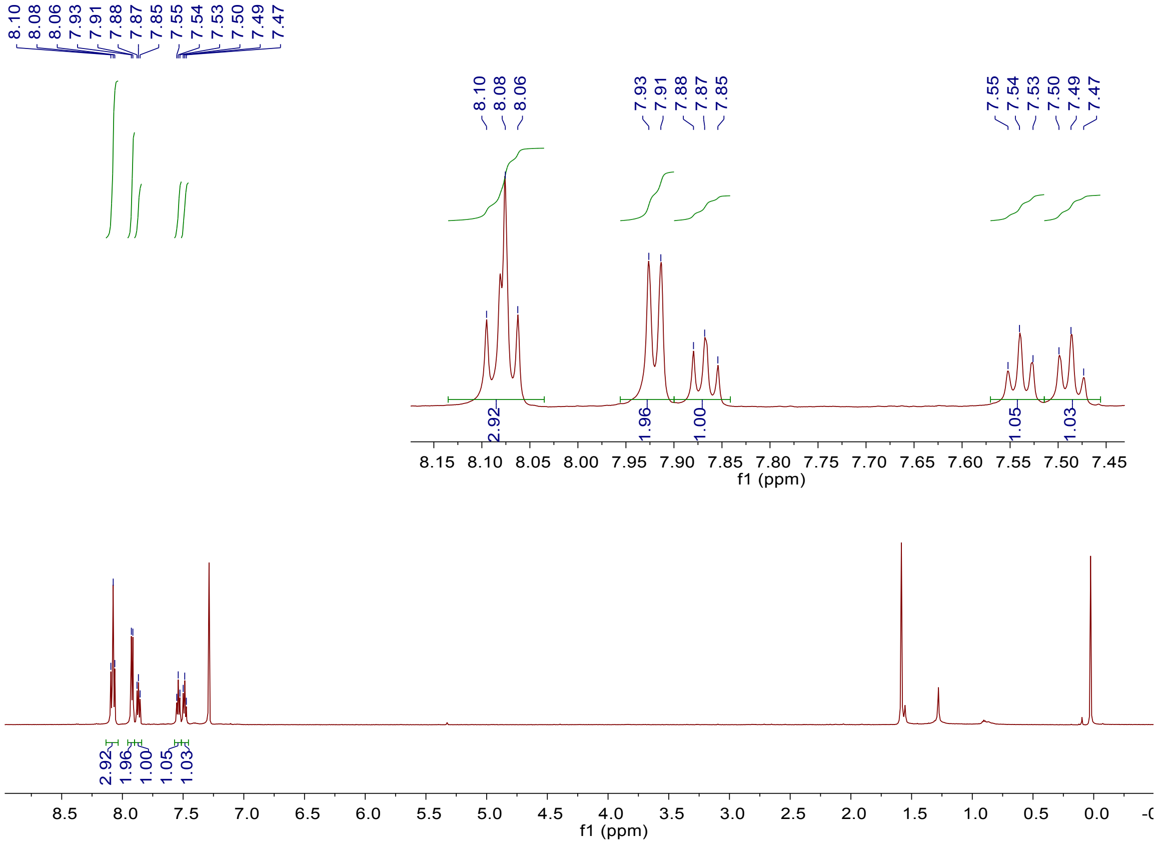

Figure S21. The ^{1}H NMR spectrum of 2,3-dicyano-fluoranthene, conducted in Chloroform-*d*.

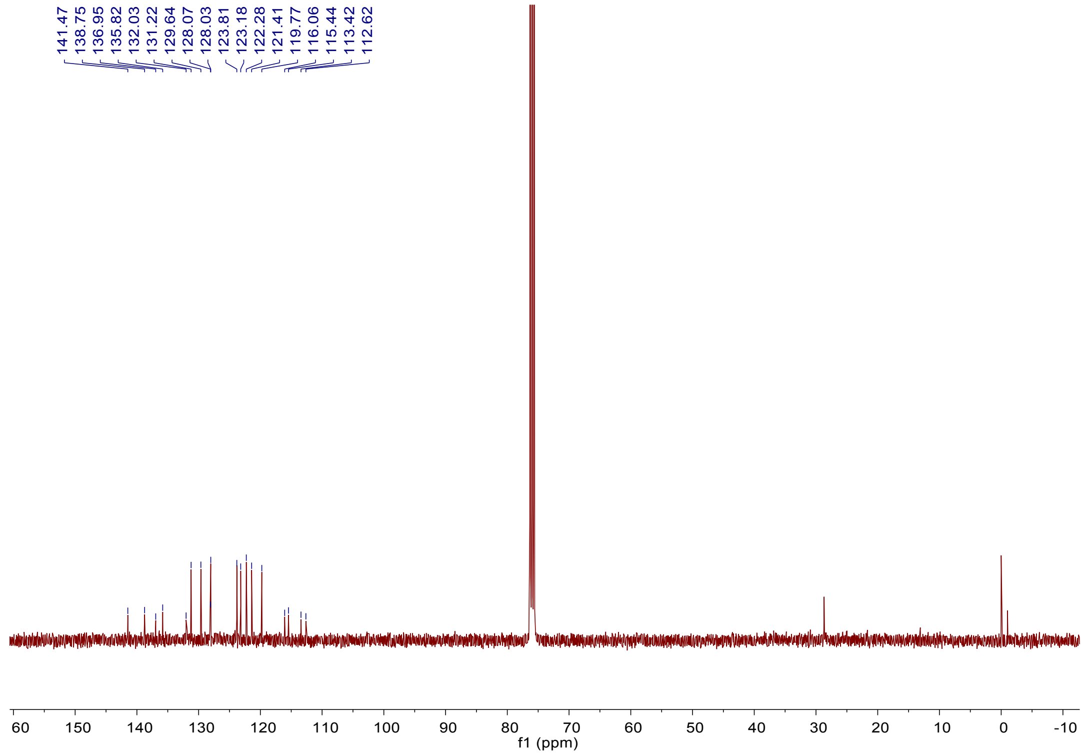

Figure S22. The ^{13}C NMR spectrum of 2,3-dicyano-fluoranthene, conducted in Chloroform-*d*.

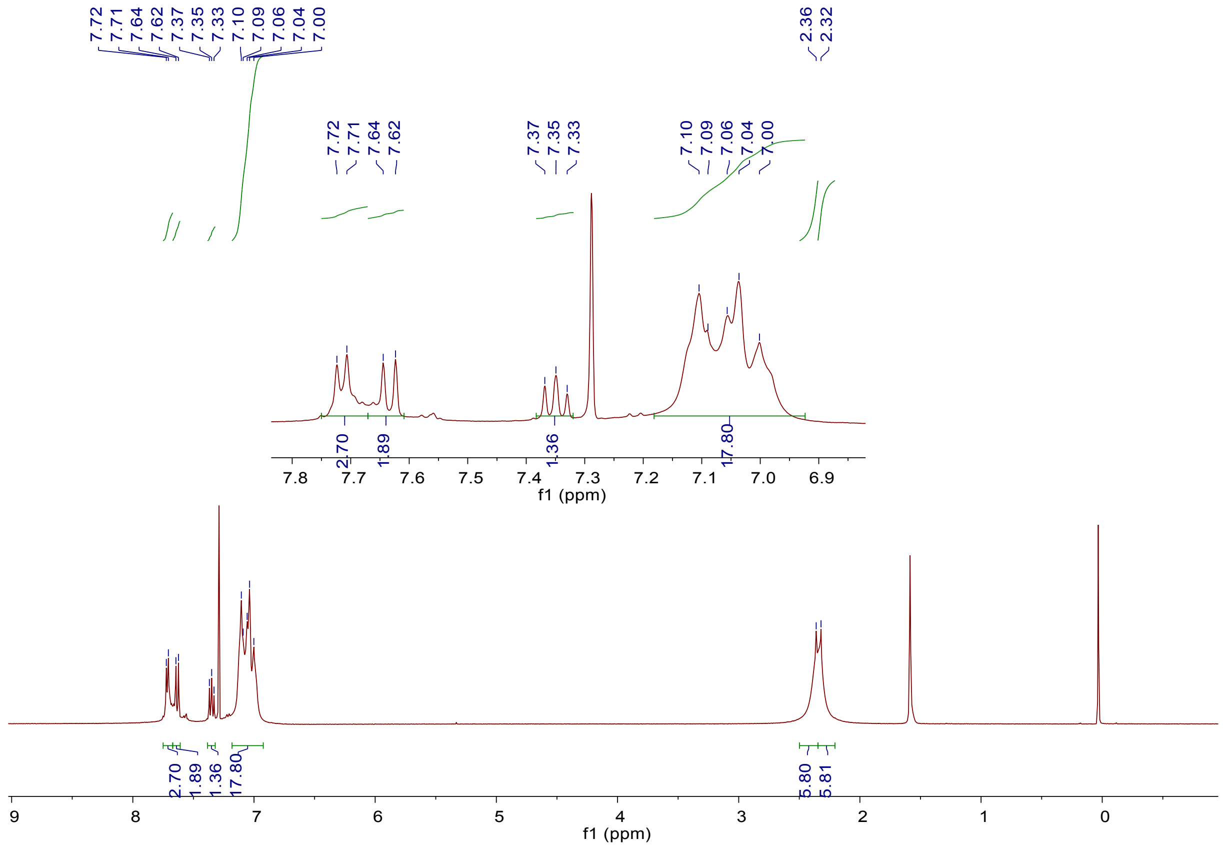

Figure S23. The ^{1}H NMR spectrum of **BTF1**, conducted in Chloroform-*d*.

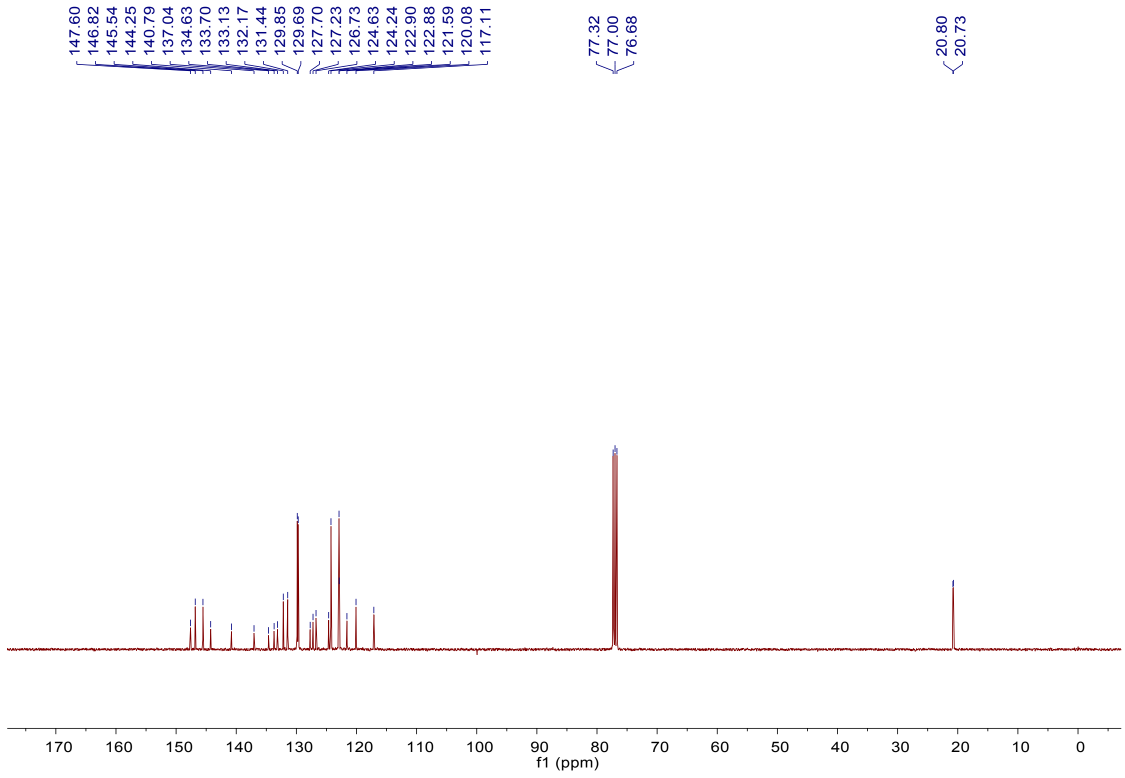

Figure S24. The ^{13}C NMR spectrum of **BTF1**, conducted in Chloroform-*d*.

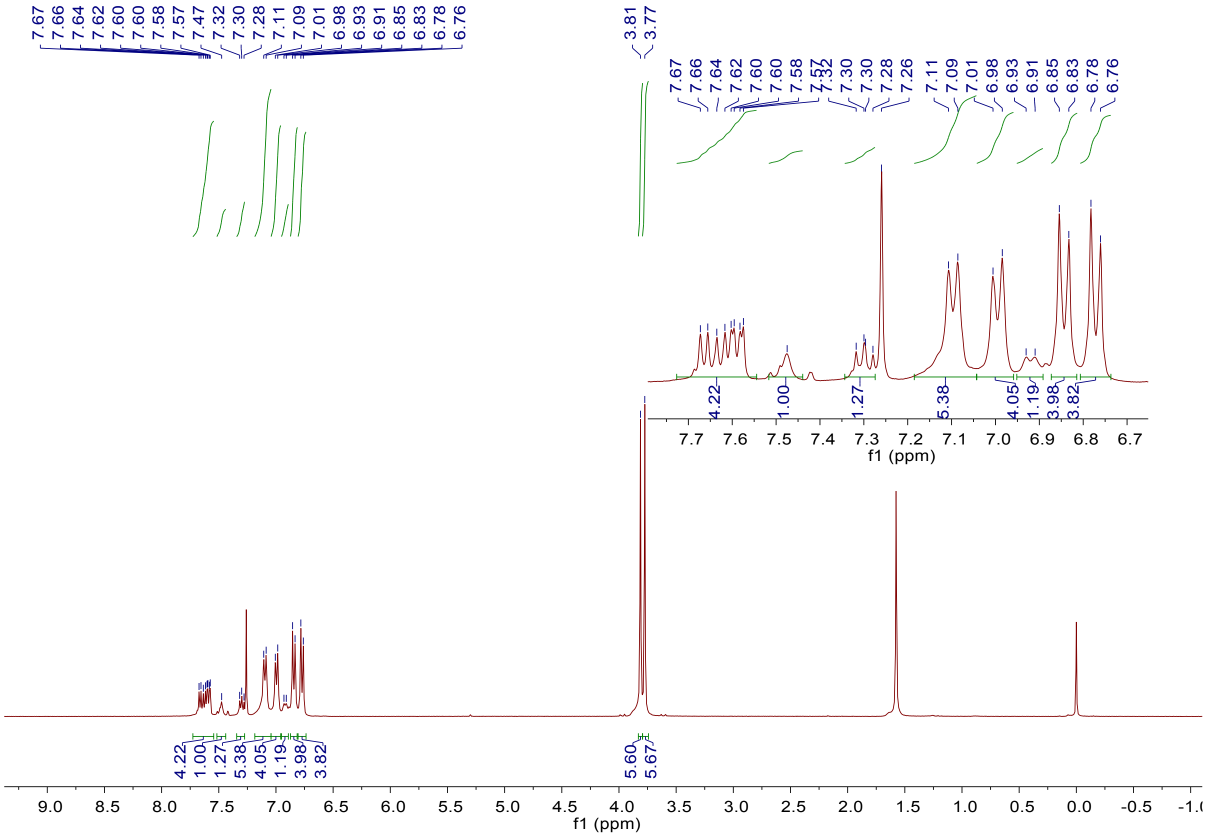

Figure S25. The ^{1}H NMR spectrum of **BTF2**, conducted in Chloroform-*d*.

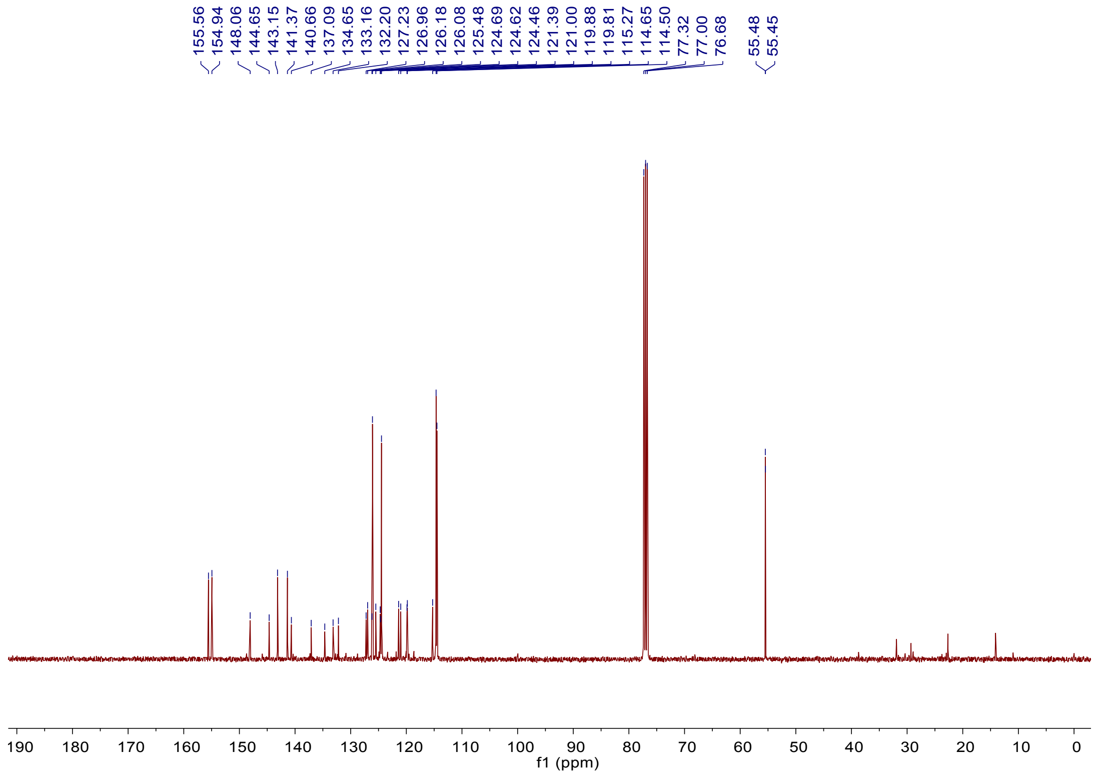

Figure S26. The ^{13}C NMR spectrum of **BTF2**, conducted in Chloroform-*d*.

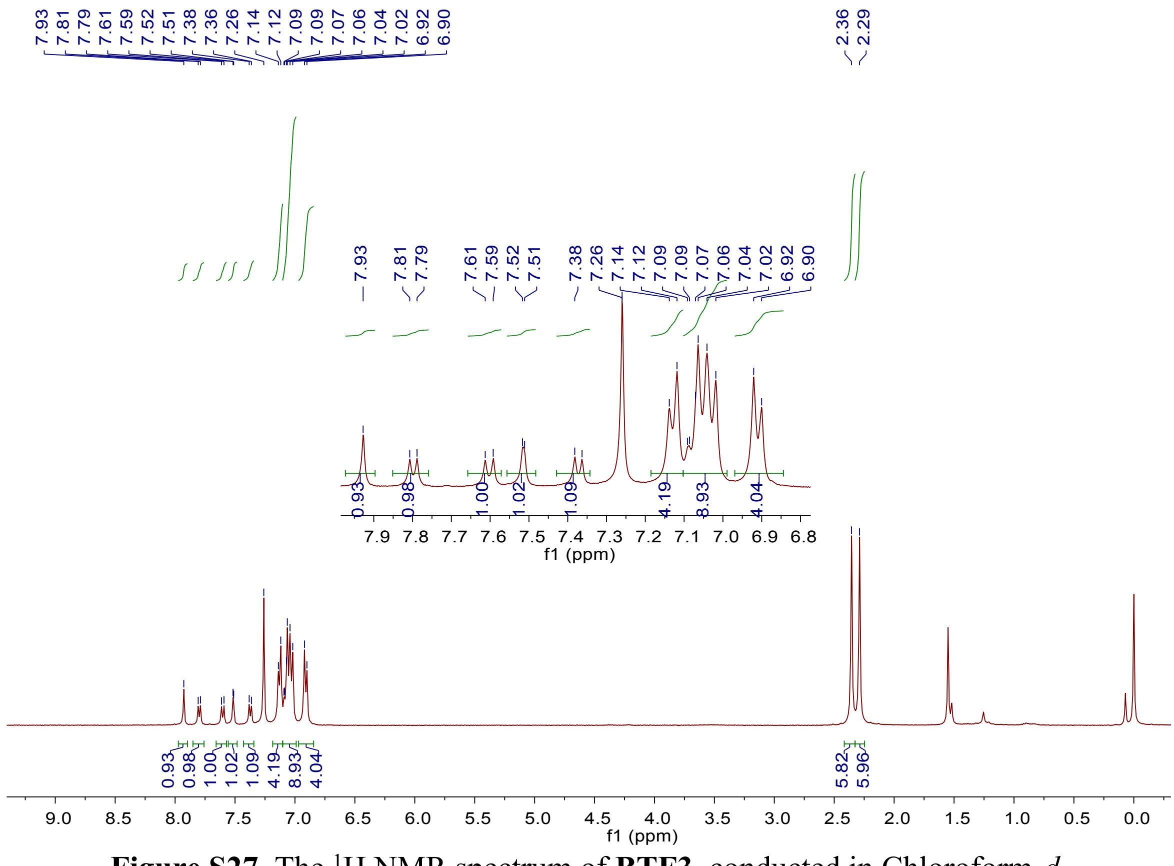

Figure S27. The ^{1}H NMR spectrum of **BTF3**, conducted in Chloroform-*d*.

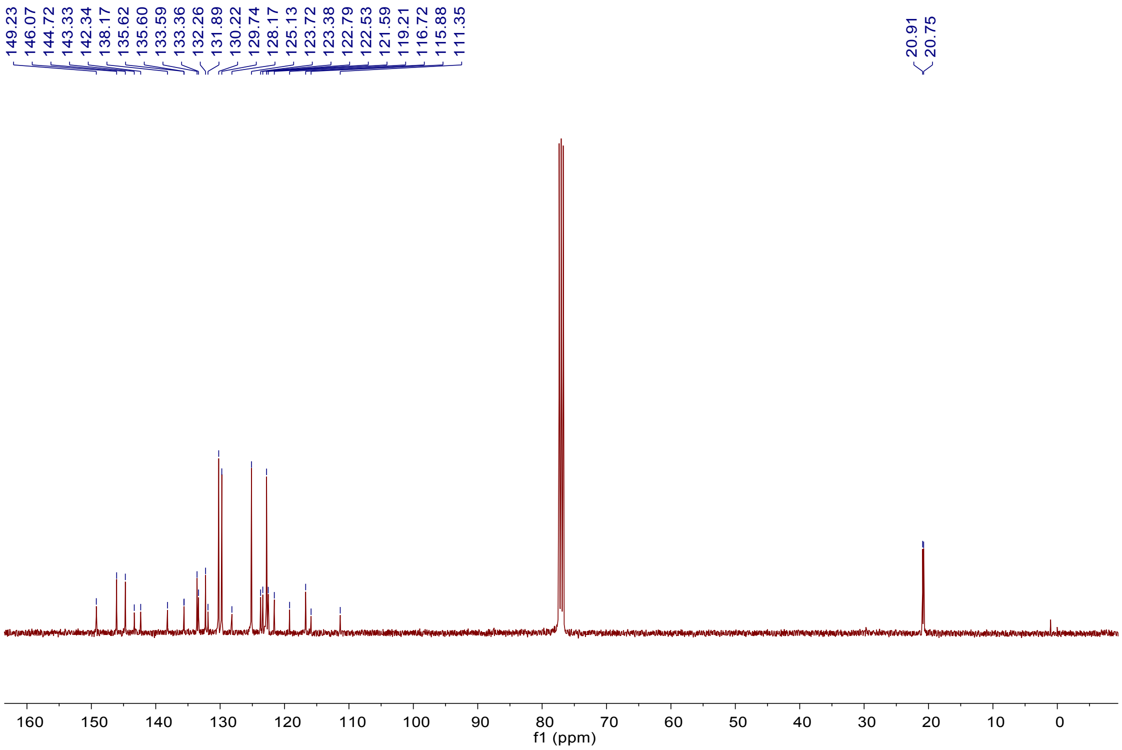

Figure S28. The ^{13}C NMR spectrum of **BTF3**, conducted in Chloroform-*d*.

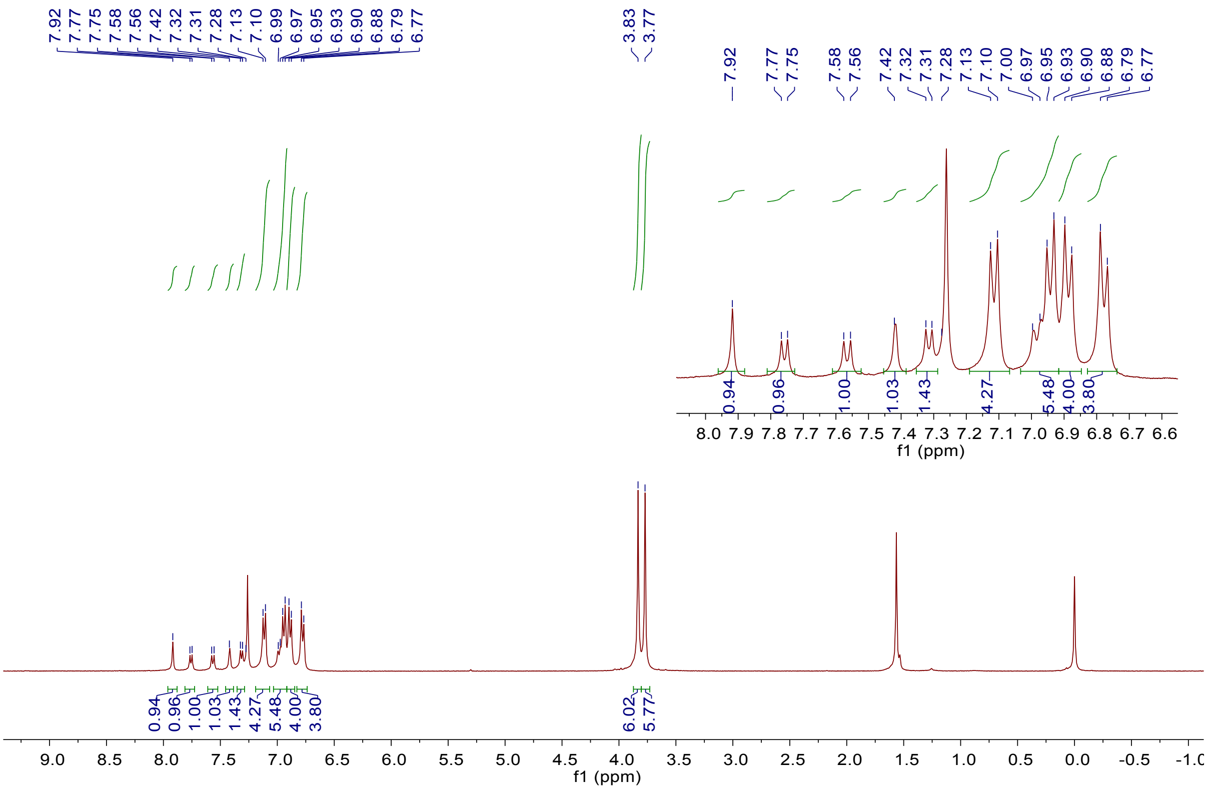

Figure S29. The ^{1}H NMR spectrum of **BTF4**, conducted in Chloroform-*d*.

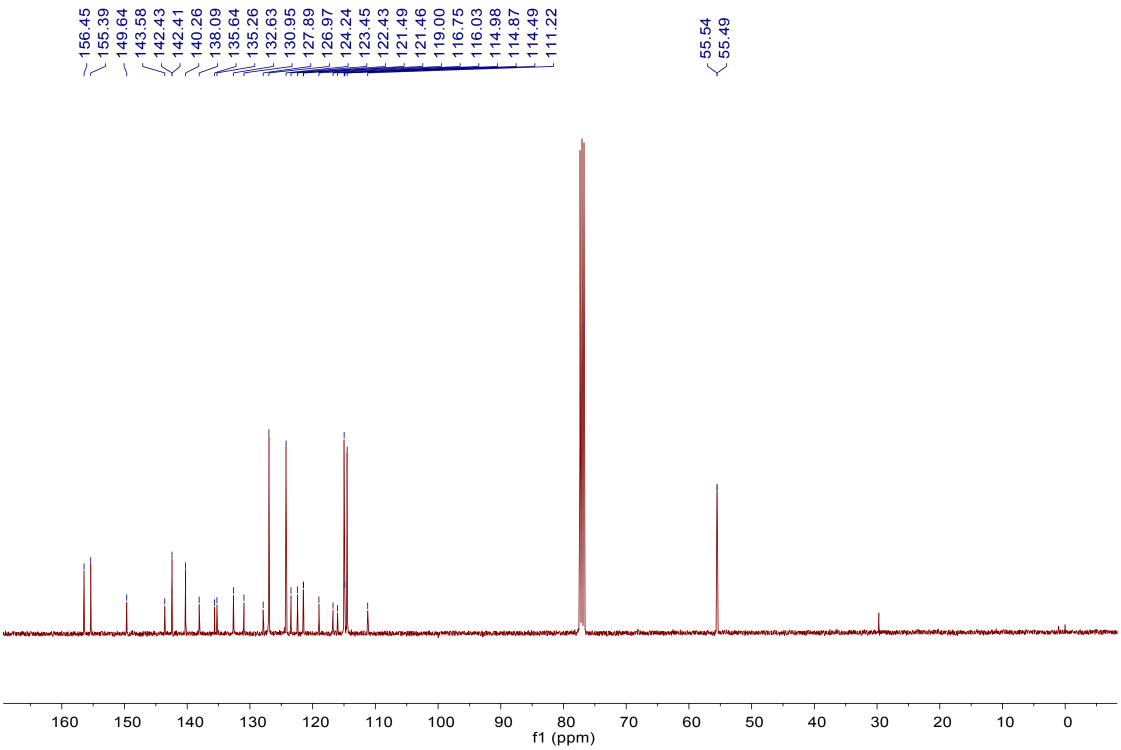

Figure S30. The ^{13}C NMR spectrum of **BTF4**, conducted in Chloroform-*d*.

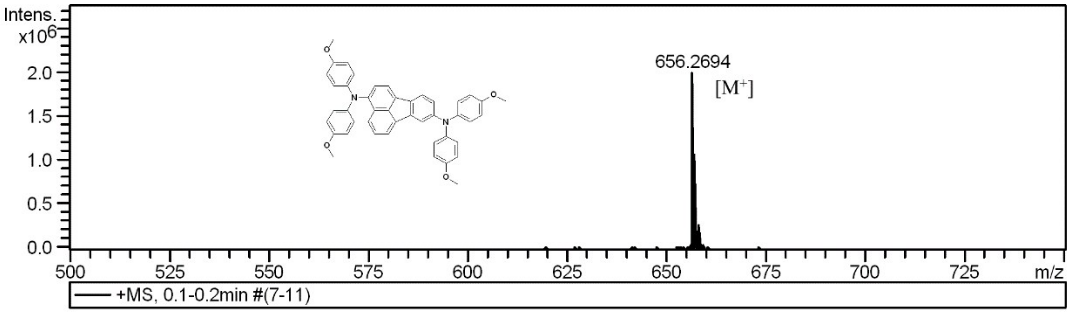

Figure S31. HR-Mass spectrum of **BTF1**.

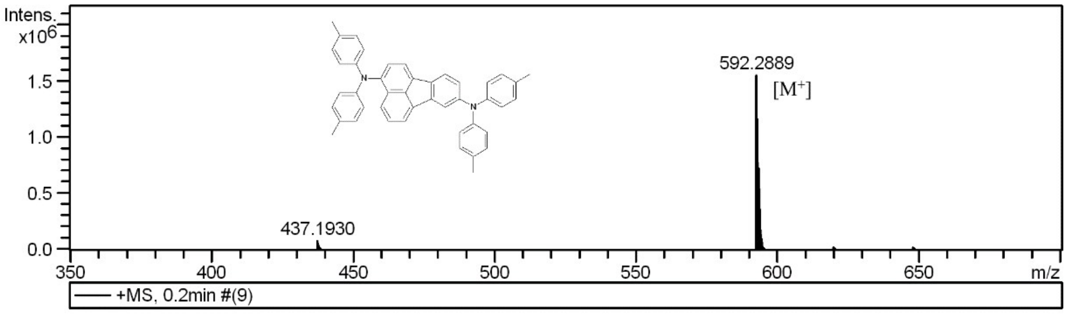

Figure S32. HR-Mass spectrum of **BTF2**.

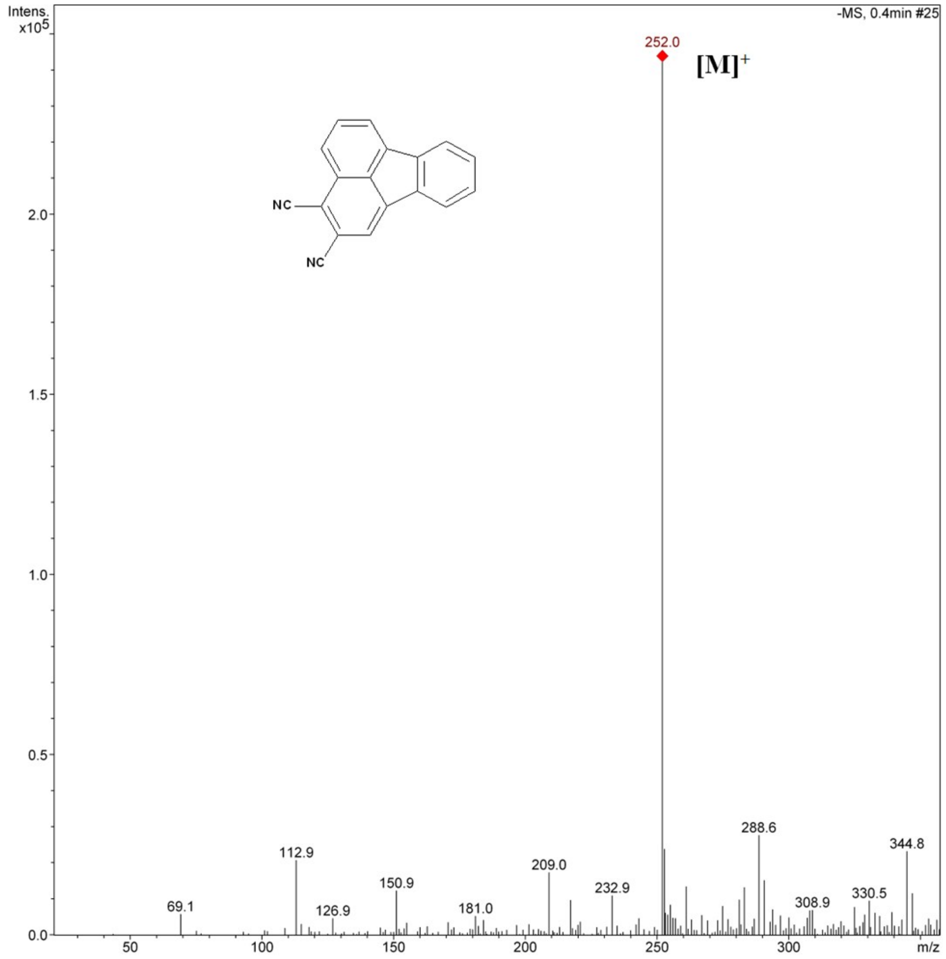

Figure S33. Mass spectrum of 2,3-dicyano-fluoranthene.

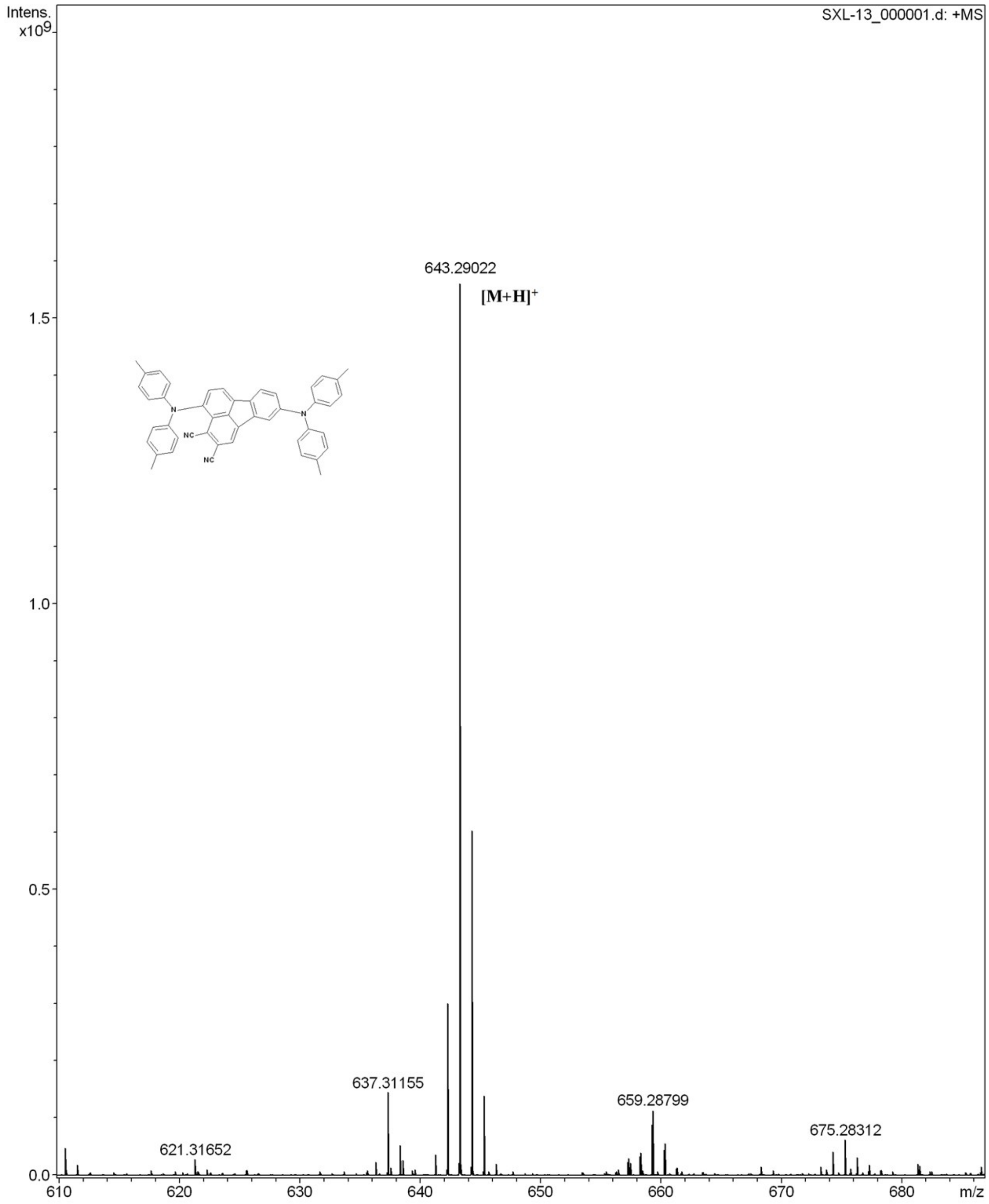

Figure S34. HR-Mass spectrum of **BTF3**.

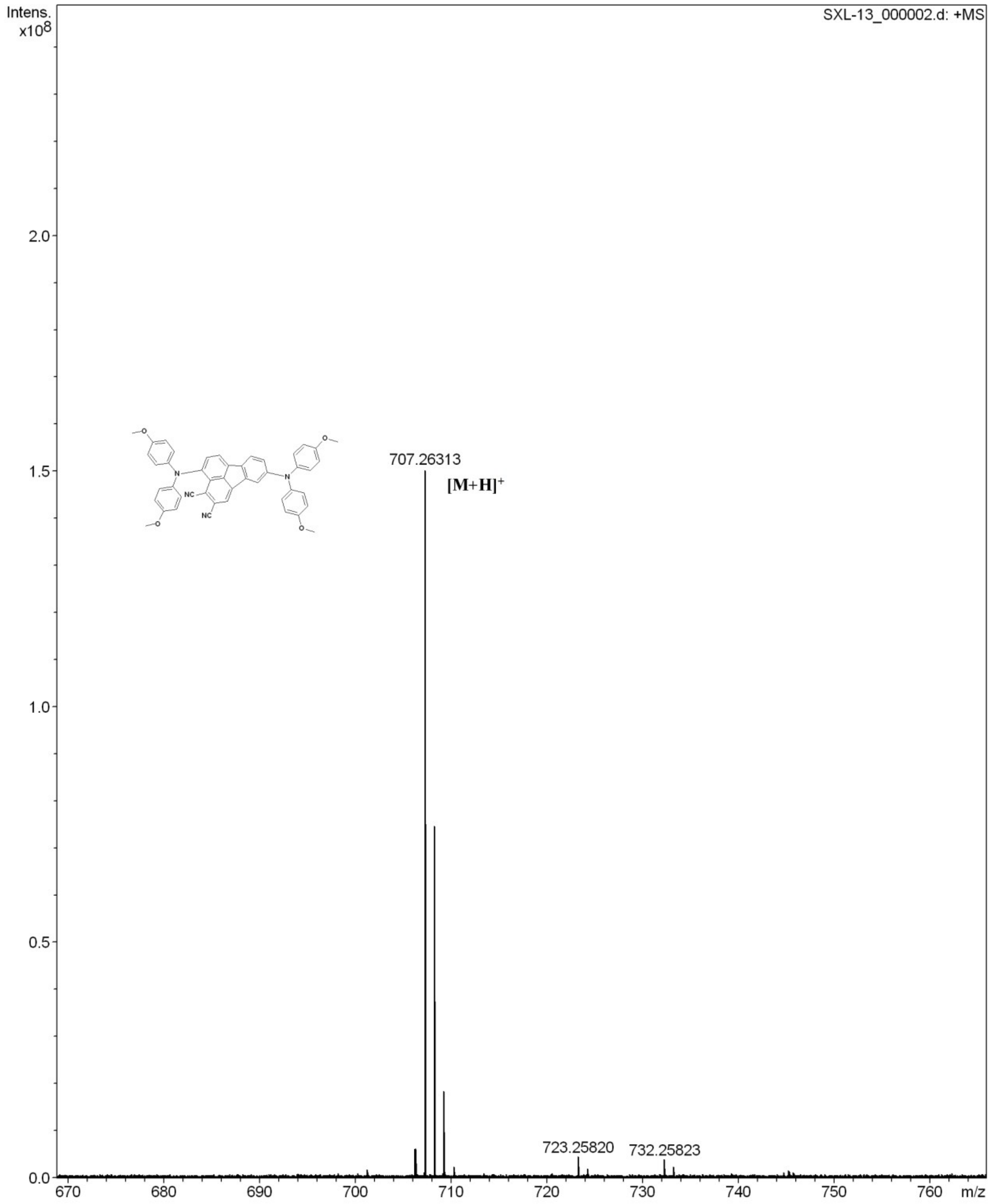

Figure S35. HR-Mass spectrum of **BTF4**.

S.8 References

[1] N. Nakano, O. Nakagawa, T. Yade, Y. Okamoto, *Macromolecules* **2003**, *36*, 1433.

[2] W. Weishi, W. Gao, **2016**, US 2016/0268514 A1.

[3] L. Macor, M. Gervaldo, F. Fungo, L. Otero, T. Dittrich, C.-Y. Lin, L.-C. Chi, F.-C. Fang, S.-W. Lii, K.-T. Wong, C.-H. Tsai, C.-C. Wu, *RSC Advances* **2012**, *2*, 4869.
